# From Surface Buffering to Bulk Ion Exchange: Mapping the Acid Response of Potassium Poly(Heptazine Imide) (K-PHI)

**DOI:** 10.1021/acs.langmuir.6c03166

**Published:** 2026-09-28

**Authors:** Julia Sinisi, Gabriel A. A. Diab, Titus Ekabat, Sebastian Bette, Viola Duppel, Ivo F. Teixeira, Stefan Repp, Bettina V. Lotsch

**Affiliations:** † Nanochemistry Department, Max Planck Institute for Solid State Research, Heisenbergstr. 1, 70569 Stuttgart, Germany; ‡ Department of Chemistry, Ludwig Maximilian University of Munich (LMU), Butenandtstr. 5-13, 81377 Munich, Germany; § Department of Chemistry, 67828 Federal University of São Carlos, 13565-905 São Carlos, SP, Brazil; ∥ Department of Physics and Materials Science, Maseno University, Private Bag, 40105 Maseno, Kenya

## Abstract

Potassium poly­(heptazine imide) (K-PHI) is a promising material for photocatalysis and energy storage, yet its application in aqueous media is limited by protonation. Cation exchange to protonated poly­(heptazine imide) (H-PHI) causes structural and optoelectronic changes. Herein, a systematic study of its acid stability regime and protonation threshold is presented through simultaneous consideration of PHI’s bulk, interface, and solution properties. Down to pH 3.0, solutions are buffered by proton adsorption while the particle bulk retains structural integrity, pointing to coexistence of H^+^ at the surface and K^+^ in the bulk. Within this regime down to pH 4.0, buffering coincides with a stable zeta potential, consistent with proton adsorption balanced by counterions. At yet lower pH, the buffer limit (pH 3.0) is characterized by surface destabilization, suggesting proton excess. At pH 2.0, protonation proceeds via bulk ion exchange and H-PHI formation.

## Graphical abstract

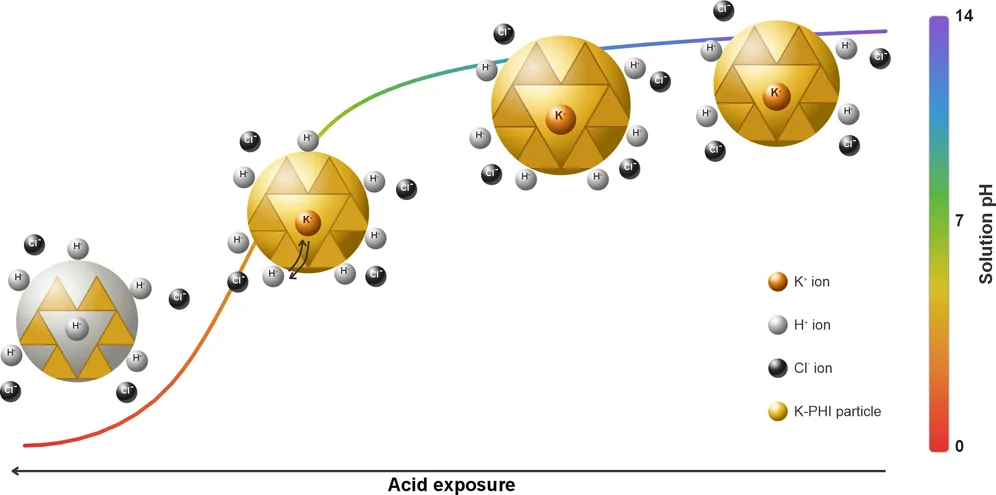

## Introduction

Carbon nitrides were first synthesized by Berzelius, Wöhler and Liebig in the 19th century, but remained largely unexplored until the 21st century,^1–4^ when advances in characterization techniques and their potential in photocatalysis and energy storage reignited interest.^5–10^ One promising subclass is constituted by metal poly­(heptazine imides) (M-PHI), consisting of heptazine units (C_6_N7) linked by imide bridges and charge-balanced by incorporated metal cations.^1,11,12^ These units form extended 2D sheets with trigonal pores accommodating the ions in their hydrated form.^11–13^ M-PHI can be synthesized either by condensing N-rich precursors with alkaline metal salts,^13–15^ or by alkaline-salt mediated restructuring of melon.^8,16^ M-PHI is insoluble in water, but the metal ions in the pores are readily exchangeable in aqueous dispersion.^15,17–21^ In the presence of acids, M-PHI loses its color^1,17,22^ and turns white, converting into protonated poly­(heptazine imide) (H-PHI). This process involves the replacement of metal cations by protons, leading to the formation of covalent N–H bonds at imide-bridging nitrogen sites of the heptazine framework.^12,13,15,17^ The overall reaction can be described by a proton-transfer equilibrium:

$${\mathrm{M‐PHI}}_{\left(s\right)}+H_{3}O_{\left(\mathrm{aq}\right)}^{+}⇋{\mathrm{H‐PHI}}_{\left(s\right)}+M_{\left(\mathrm{aq}\right)}^{+}+H_{2}O_{\left(l\right)}$$

The reversible exchange points to K-PHI and H-PHI as a conjugated Brønsted acid–base pair: K-PHI can accept protons from aqueous solution, while H-PHI can donate them. This behavior exemplifies the general principle that acid–base sites on solids largely mirror those in solution.^23–26^ Well-known representatives of solid acids include zeolites, mixed metal oxides, and oxo salts.^26^ The strength of such solid acids and bases are commonly assessed by solution-phase titrations with Hammett color indicators under dry or humidity-controlled conditions, or by temperature-programmed desorption of adsorbed bases such as ammonia and pyridine.^24,25,27^ The Hammett acidity function, H_0_, is often used to derive p*K*_A_-type values, but we note that H_0_ is defined for homogeneous acidic solutions^25^ and may be ambiguous for porous solids like PHI with limited site accessibility.^23^

While the ion exchange of M-PHI itself is well-established, especially for K-PHI, the experimental protocols (particularly acid concentration and exposure time) vary significantly,^12,13,20,28,29^ resulting in differing reported structures and properties of H-PHI.^12,13,15,30,31^ These differences reflect the sensitivity of the system to the applied conditions. However, the steps eventually leading to ion exchange remain poorly characterized. PHI has been described as a weak acid while being fully stable in aqueous media,^15^ i.e., without partial dissociation common for weak bases. Alternatively, replacement of K^+^ by H^+^ has been described as gradual and initially linear with an increase in acid concentration.^32^ As a result, the behavior in aqueous media and thus the precise stability window relevant for applications remains insufficiently defined. The recovery of M-PHI from H-PHI has been demonstrated.^15,28^ However, incomplete reversibility suggests that protonation under harsh conditions may alter the structure beyond simple ion-exchange.^13^

To address this, we systematically investigate the behavior of one example of K-PHI (synthesized according to Schlomberg et al.^13^) in aqueous dispersions across the entire pH range. We consider the interplay between particle bulk, interface, and solution. A controlled experimental design exposes K-PHI to defined pH environments while maintaining otherwise constant conditions, namely solution volume and equilibration time. Varying the pH while maintaining a constant dispersion volume (i.e., without dilution) ensures that the solid concentration and, consequently, the number of PHI protonation sites per volume remain unchanged. Proton interaction is thus governed by proton availability (i.e., solution pH). Applying identical equilibration times at each pH further isolates proton concentration as the only varying parameter. Additionally, we apply Le Chatelier’s principle to assess whether the presence of additional K^+^ cations in solution—and hence, ionic strength—enhances the resistance to protonation. Through detailed characterization of the particle bulk, interface, and surrounding solution we aim to map the acid stability pathway, identify critical thresholds, and clarify the role of ion exchange vs surface ion adsorption.

## Results and Discussion

As a benchmark experiment, K-PHI (TEM and FFT inset of the polycrystalline starting material shown in Figure 1a) was titrated with both a strong acid (1.0 M HCl) and strong base (1.0 M KOH), as shown in Figure 1b,c (photographs of the titration series are provided in Figures S1 and S2). K-PHI, initially dispersed in neutral ultrapure water (18.2 MΩ·cm), forms alkaline dispersions of pH 10.8 (pH measurements were conducted using a Mettler Toledo pH meter). Initially, upon addition of either HCl or KOH (each up to 20 μL in 20 mL titrand), the pH remains stable, suggesting buffering behavior. This plateau corresponds to an apparent p*K*_B_ of 3.2 for K-PHI. Here, the use of the Henderson–Hasselbalch equation, which is strictly defined for homogeneous solutions only, serves as an approximation. For solid acids, the Henderson–Hasselbalch approach suffers from distinct limitations. Examples include the approximation of activities by concentrations and the assumption of established equilibrium conditions, particularly when proton exchange within the solid bulk is slow.^33^ Accordingly, the obtained apparent p*K*_B_ does not represent a thermodynamic p*K* value, rather an approximate descriptor of the observed buffer behavior. With increasing acid addition, the acid titration curve eventually adopts an S-shaped profile. An apparent equivalence point is observed at pH 6.6, beyond which complete conversion to H-PHI is expected. The equivalence point lies slightly below pH 7, as is characteristic for weak bases, and as previously noted in literature.^15^ Figure S6 further shows the acid titration curves for different K-PHI concentrations (ranging from 10 mg to 100 mg in 20 mL dispersions; visually illustrated using the pH indicator phenolphthalein in Figure S3), highlighting that K-PHI’s acid response depends on the solid concentration in dispersion. Here, the buffer capacity increases with increasing K-PHI concentration.

**Figure 1:**
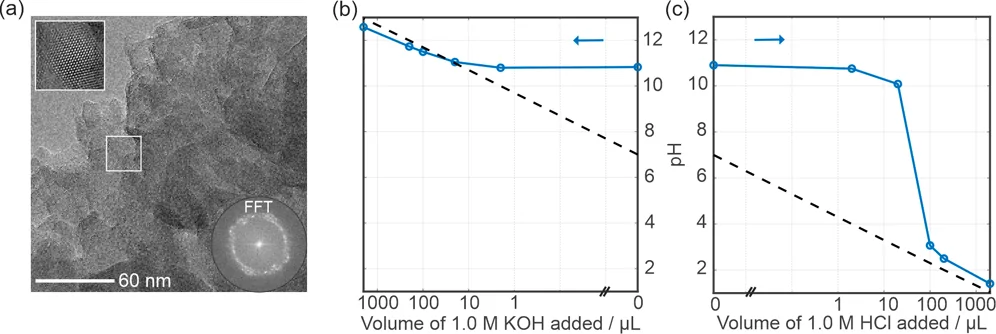
(a) TEM image of the starting material K-PHI (scale bar: 60 nm). The rectangular inset in the top left shows a magnified view of the boxed region, while the circular inset shows the calculated Fast Fourier transform (FFT). (b) Alkaline titration of K-PHI with 1.0 M KOH and (c) acidic titration with 1.0 M HCl.

Following the titration experiment, the acid exposure series was performed by dispersing 150.0 mg of K-PHI in preadjusted aqueous solutions of varying acidity (60 mL final volume to avoid dilution effects) and analyzed after 30 min of equilibration (Figure 2). Based on complementary characterization of the particle bulk, interface, and surrounding solution, this series identifies a distinct three-step acid response of K-PHI: Consistent with the titration results, the K-PHI dispersion maintains a nearly constant solution pH of 11.1 even upon acid exposure equivalent to pH 3, reflecting effective buffering of the acid (Figure 2a, input data listed in Table S1, molar quantities plotted in Figure S5 as protons dissolved vs. protons added). The compositional analysis of solid particles reveals a slight reduction in potassium content already upon dispersion in neutral water, indicating minor potassium leaching during simple dispersion in water. However, upon exposure to acidic conditions within the buffering regime (neutral to pH 3.0), the potassium content of the bulk particles remains constant (Figure 2b), suggesting that proton uptake is not accompanied by potassium release. In line with this, Fourier-transform infrared spectroscopy (FT-IR) spectra confirm that potassium remains coordinated to bridging imide sites (Figures S10–S14).^1,15,34^ In parallel, both hydrogen and water content of the solid remain nearly constant (Figures S8a and S9), ruling out a significant proton or water incorporation into the layered structure. This behavior—K-PHI maintaining both constant potassium and hydrogen content—indicates that the observed proton removal from solution cannot be attributed to bulk uptake of protons or bulk ion exchange with potassium, but must be localized at the particle surface. The finding that protons are removed from solution while potassium remains in the lattice points to a coexistence of H^+^ and K^+^, with adsorbed protons residing at the surface while lattice potassium ions remain bound in the bulk.

**Figure 2:**
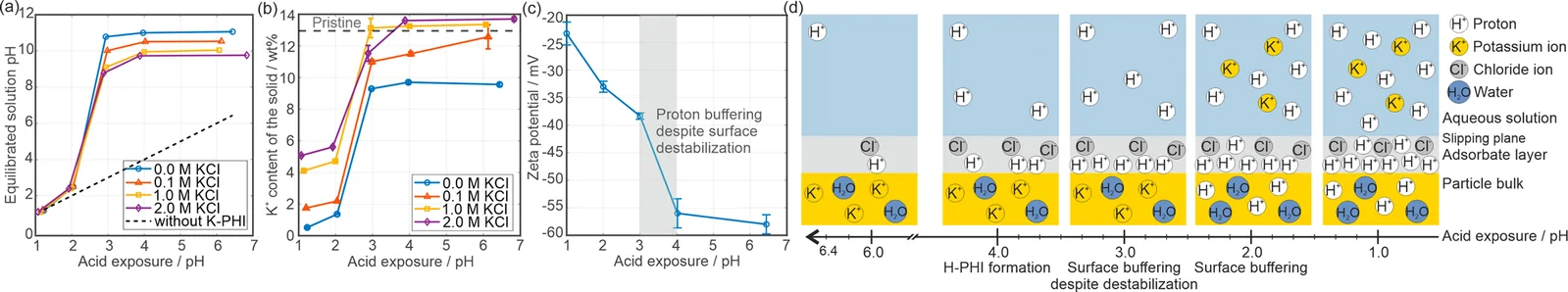
Comparison of (a) the pH response of the solution, (b) the potassium content of the solid particle, and (c) the zeta potential at the particle surface of K-PHI aqueous dispersions as a function of acid exposure. Error bars in (b) and (c) correspond to the standard deviation of three independent measurements and are smaller than the symbol size where not visible. (d) Schematic summary of the proposed buffering behavior of K-PHI in KCl-free dispersion and the formation of H-PHI under increasing acidity: Buffering is sustained by a proton adsorbate layer initially electrostatically compensated by counterions, and maintained even as the balance shifts to proton dominance (pH 3.0). At a proton breakthrough of pH 2.0, the buffering capacity collapses, leading to bulk ion exchange and H-PHI formation. Ion populations are shown conceptually.

Zeta potential measurements further resolve the surface buffering behavior into two regimes. The first is characterized by a nearly constant electrostatic potential across the initial buffering region, between pH 6.4 (−58 mV) and pH 4.0 (−56 mV; Figure 2c). A possible explanation is proton adsorption being balanced by negatively charged counterions from the surrounding solution, such as OH^–^ and, especially at lower pH, increasingly Cl^–^, preventing net charge accumulation. Similar behavior has been observed in solid acids and bases that buffer the pH of the surrounding solution by accepting or donating H^+^ ions together with Cl^–^.^35–37^

A characteristic feature of the buffering regime is its sensitivity to the ionic strength of the solution (Figure 2a), a behavior mostly documented for dissolved buffer systems.^38,39^ For K-PHI dispersions, higher KCl-salt concentrations shift the adsorption equilibrium toward more free protons in solution. Consequently, the base strength of K-PHI is reduced, reflected in an increase in the apparent p*K*_B_ from 3.2 without KCl, to 3.5 at 0.1 M KCl, 4.0 at 1.0 M KCl, and 4.2 at 2.0 M KCl. This is attributed to increased ionic strength, which lowers the proton activity and compresses the electric double layer^40^ as reflected in the decreasing Debye–Hückel screening length (calculated to 0.96 nm for 0.1 M KCl, 0.30 nm for 1.0 M KCl, 0.22 nm for 2.0 M KCl).^41^ Consequently, the alkaline buffering behavior of K-PHI dispersions can be tuned by adjusting the salt concentration. Crucially, these KCl-induced changes in the solution phase within the buffer region are not reflected in the bulk composition and structure of the solid particles (Figure 2b, Figures S10–S14, Figures S20–S24), reinforcing the structural integrity of the solid particles across the buffering range. Prior to analysis, particles were ethanol-washed to remove only surface-adherent salt residues while avoiding alteration of preceding experiment results, such as additional solvent-induced leaching of potassium from within the particle bulk (Table S4, Figures S16 and S26).

The second acid response step, resolved through zeta potential measurements, occurs between pH 4.0 to pH 3.0, marking the limit of the buffering regime. Although buffering persists (Figure 2a), the zeta potential no longer remains constant but clearly becomes less negative from −56 mV to −38 mV (Figure 2c, gray area), indicating a loss of electrostatic stabilization. A pronounced increase in zeta potential across the transition from pH 4.0 to 3.0 is consistent with literature reports.^42,43^ This behavior may reflect a regime in which continued proton adsorption eventually exceeds compensation by counterions, leading to a net decrease in electric potential at the slipping plane. Elemental analysis (EA) evidence constant hydrogen content of the solid bulk despite surface destabilization (constant 7.2 H per heptazine unit in the range between pH 6.4 to pH 3.0; Table S3, Figure S8a). Simultaneously, inductively coupled plasma atomic emission spectroscopy (ICP-AES) shows a constant potassium content (Figure 2b), while FT-IR analyses confirm intact K^+^–imide interactions (Figure S11),^1,15,34^ indicating that no ion exchange has yet occurred.

Further acidification to pH 2.0 pushes the system beyond its buffering capacity corresponding to the third acid response step, characterized by a marked shift in solution pH (Figure 2a). The electrostatic imbalance present at pH 3.0, is relieved by bulk protonation at pH 2.0, as evidenced by bulk analysis. The potassium interaction with the bridging imide dissociates abruptly (Figures S10–S15), leading to a sharp decrease in potassium content by 0.64 (±0.01) K^+^ per heptazine unit compared to K-PHI dispersed in ultrapure water (Figure S8a). This loss is driven by the incorporation of 0.64 (±0.02) H^+^ per heptazine unit (Figure S5), marking the conversion of K-PHI to H-PHI.^1,15,34^ The transition is abrupt and independent of the potassium concentration in solution, consistent with the fact that only a limited number of structural sites in the bulk material can be protonated. Thermogravimetry (TG) and EA data substantiate a protonation threshold, showing a concomitant rise in total hydrogen content at pH 2.0 along with a 1.5-fold increase in water content compared to K-PHI in ultrapure water (Figures S8 and S9). The transition is accompanied by a decrease in water desorption temperature (Figure S9c), consistent with the weaker pore–water interactions characteristic of H-PHI.^13^ Residual potassium in H-PHI increases with the KCl concentration used during acidification (Figure 2b). This indicates that although protonation is complete, as evidenced by fully formed N–H bonds, steric hindrance and slow diffusion results in residual K^+^ in the particle bulk. Protonation is reflected in concurrent changes to the surface properties, evidenced by an increase in zeta potential from −38 mV to −33 mV upon lowering the pH from pH 3.0 to pH 2.0 (Figure 2c). A less negative zeta potential is consistent with protonation of the framework at negatively charged imide sites formerly coordinated to K^+^, while an overall still negative potential is consistent with literature.^42,44^

Taken together, solution acidity, bulk composition, and surface electrostatics (Figure 2a–c) identify a three-step acid response of K-PHI, schematically summarized in Figure 2d. Buffering persists upon acid exposure down to pH 3.0, associated with proton adsorption while preserving the structural integrity of K-PHI. Within this region, down to pH 4.0, this buffering coincides with an unchanged electrostatic potential at the slipping plane, consistent with proton adsorption accompanied by counterions. Approaching the buffer limit between pH 4.0 and 3.0, the surface shifts toward electrostatic imbalance, pointing to predominant proton adsorption. Further acidification to a critical pH 2.0 eventually induces protonation via bulk ion exchange and H-PHI formation. The overall processes may be approximated by the idealized reaction Scheme 1.

**Scheme 1:**
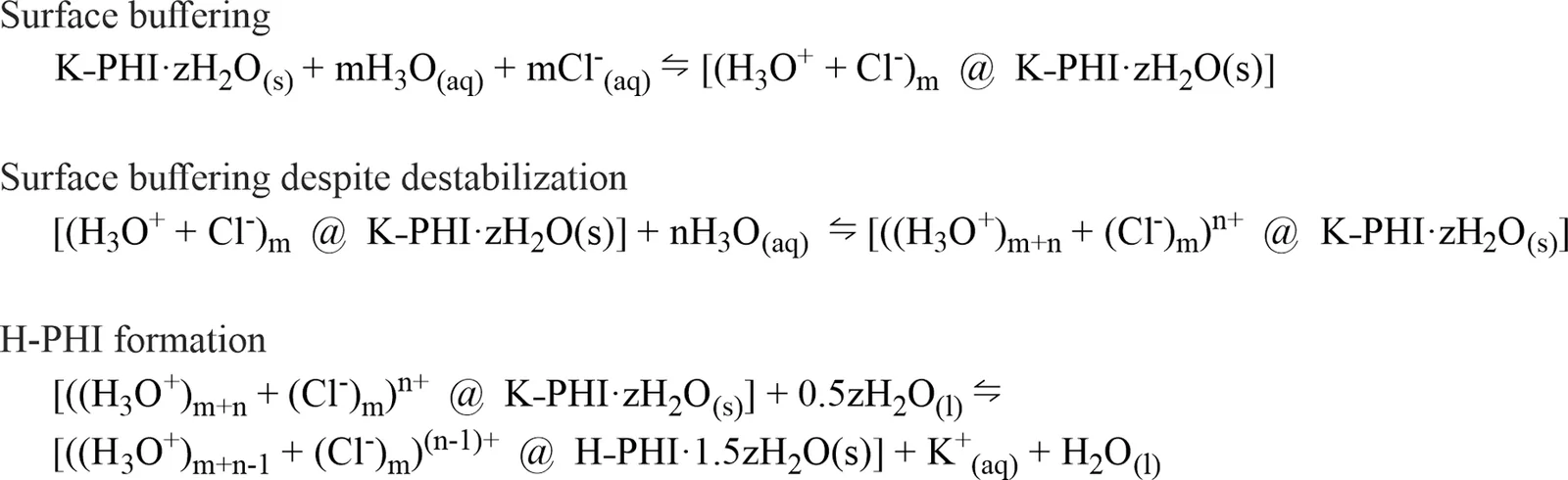
Proposed K-PHI Buffering in Acidic Aqueous Dispersions *via* Proton Adsorption, Initially Maintaining an Unchanged Electrostatic Potential up to the Slipping Plane. Surface Destabilization, Potentially due to Surface Proton Excess, Precedes Bulk Protonation at pH 2.0

Particular attention is given to the polymer obtained at pH 2.0, representing the mildest conditions for H-PHI generation from K-PHI, involving minimal protonation beyond ion exchange. The following discussion focuses on the sample obtained from the 0.0 M KCl series (i.e., pH 2.0, 0.0 M KCl), facilitating comparison with previous literature reports. Under these conditions, K^+^–imide interactions are replaced by covalent N–H bonds, marking a shift from ionic to covalent bonding. FT-IR (Figures S10–S15) confirms full protonation of bridging imides (N–H stretching at 3450 cm^–1^, deformation bands at 1580 and 1550 cm^–1^), accompanied by an abrupt disappearance of the K^+^–imide interaction (1145 and 989 cm^–1^),^19,45^ regardless of potassium concentration in solution. Cyanamide groups undergo weak but progressive degradation with decreasing pH (Figures S10c, S12c, S13c, S14c, and S15c), without the formation of −NH_2_ bands, which would indicate hydrolysis to urea rather than simple protonation. Yet, no changes in bulk composition are observed (no EA shifts; Table S3), suggesting that protonation remains surface-specific. This underscores the necessity of using the mildest possible conditions for H-PHI preparation to avoid degradation of cyanamide groups. Solid-state nuclear magnetic resonance (ssNMR; Figure S18), with assignments guided by computational models from previous studies,^13,46–48^ reveals a reduction of the ^13^C spectrum from five to two signals, reflecting decreased local diversity due to covalently localized N–H bonds. ^1^H ssNMR shows a downfield shift of the NH signal (from 10 to 12 ppm), indicating increased acidity.^13^ X-ray diffraction (XRD; Figures S20–S25, peak positions listed in Table S5) shows an abrupt upshift of the 010 reflection starting at pH 2.0, consistent with protonation of K-PHI and analogous to ion exchange with smaller metal cations.^12^ The slight downshift of the 001 peak in H-PHI prepared from higher KCl-concentrated solutions, correlates with the residual potassium content in the particle (Figure S25), suggesting interlayer expansion by incorporated additional water molecules.^13^ Taken together, these observations summarize the protonation of K-PHI to H-PHI at pH 2.0, which is accompanied by the loss of potassium ions and a corresponding proton uptake, the formation of secondary amine functionalities at the expense of K^+^–imide interactions (FT-IR), additional water incorporation, and slightly increased interlayer spacing (XRD).

Finally, the proton–potassium exchange is confirmed to be reversible. H-PHI is regenerated by titration with 1.0 M KOH (Figure 3a, molar quantities plotted in Figure S7 as KOH dissolved vs. KOH added), in accordance with acid–base reactivity and established cation-exchange protocols for PHI. The titration reaches pH 11.6, slightly above pristine K-PHI’s pH in neutral water, ensuring complete conversion. The transition to K-PHI is accompanied by a color change from white to yellowish (Figure S4). Proton release (0.66 ± 0.02 per heptazine) matches the uptake during H-PHI formation, consistent with stoichiometric proton exchange. H-PHI dispersions show acidic behavior (apparent p*K*_A_ 4.7, derived via Henderson–Hasselbalch; the limitations discussed above apply, i.e., the apparent p*K*_A_ does not represent a thermodynamic p*K* value, which also explains the lack of complementarity with the apparent p*K*_B_ of 3.2 obtained for K-PHI (p*K*_A_ + p*K*_B_ ≠ 14)). ICP-AES, EA and TG (Tables S2 and S3, Figures S8b and S9) confirm full K^+^ recovery (0.97 ± 0.01 for regenerated K-PHI vs. 1.02 ± 0.02 K^+^ per heptazine unit for pristine K-PHI). Hydrogen and water contents show inverse trends, accompanied by increased water desorption temperatures. FT-IR (Figure S17) shows reformation of K^+^–imide interactions replacing secondary amines. Under acidic conditions degraded cyanamides are only partially recovered (75%), underscoring the need for mild conditions during H-PHI synthesis. ssNMR (Figure S18) shows reversion of ^13^C spectra to a more structured pattern, indicative of a heterogeneous potassium distribution in the pores and between the layers, while the secondary amine resonance in the ^1^H NMR spectrum (Figure 3b, full spectrum in Figure S18a) shifts upfield to its original position. Deconvolution of the ^1^H spectra (Figure S19) confirms complete deprotonation of bridging secondary amines, the bonding state of pristine K-PHI. This is further supported by Magic-angle-spinning (MAS) ssNMR ^1^H–^13^C heteronuclear correlation (HETCOR) spectra (Figure 3e–g). The XRD patterns of regenerated and pristine K-PHI are nearly identical, with the only difference being a very slight shift of the 001 peak to higher angles (28.04° in pristine K-PHI to 28.09° in regenerated K-PHI, Figure S27), indicating a minor reduction in interlayer distance.^13^ TEM (Figure 3c,d) confirms preserved flake morphology and crystallinity. Thus, the transition from H-PHI back to K-PHI is confirmed to be largely reversible. Only the acid-sensitive cyanamide groups could not be fully recovered, despite using H-PHI prepared under the mildest acidic conditions where cyanamide degradation was minimal. This may be relevant for applications such as photocatalysis, where cyanamide groups in PHI have been associated with enhanced photocatalytic activity and electrochemical charge storage capacity.^8,49^

**Figure 3:**
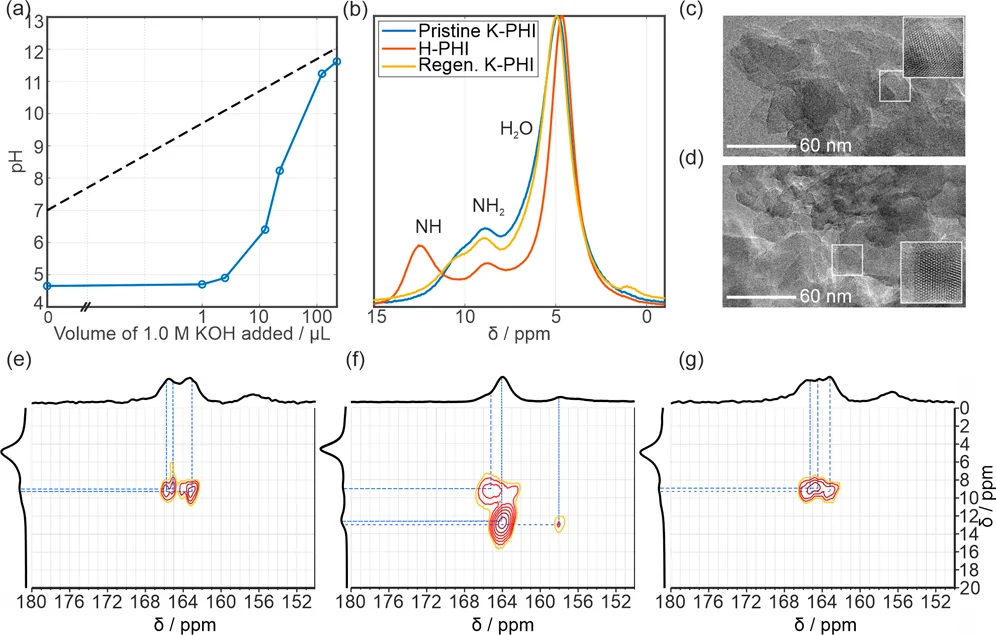
(a) Alkaline titration with KOH for targeted recovery of K-PHI from H-PHI; (b) ^1^H NMR spectrum confirming deprotonation and restored lower acidity of secondary amines in regenerated K-PHI; TEM images of (c) H-PHI and (d) regenerated K-PHI (scale bar: 60 nm). Rectangles indicate magnified regions. MAS ssNMR ^1^H–^13^C HETCOR spectra of (e) K-PHI, (f) H-PHI, and (g) regenerated K-PHI.

## Conclusion

In summary, the acid stability range and protonation mechanism of K-PHI in aqueous dispersions was systematically investigated through a three-pronged approach where particle bulk, interface, and the surrounding solution were analyzed simultaneously. K-PHI’s behavior depends on the environmental acidity: Acidic solutions are buffered by adsorption up to pH 3.0, while the bulk integrity of K-PHI is fully preserved. K-PHI thus appears to maintain a coexistence of K^+^ in the particle bulk and H^+^ at the particle surface. The buffering range is tunable by the ionic strength of the solution. While the electrostatic surface properties remain largely unchanged with increasing acid exposure down to pH 4.0, only the buffer limit (pH 3.0) is characterized by an electrostatic surface destabilization. This behavior may be attributed to an initial electrostatic compensation of adsorbed protons by counterions, followed by a shift to proton dominance. Finally, buffering collapses only at a protonation threshold of pH 2.0, which leads to bulk ion exchange and converts K-PHI fully into H-PHI under the mildest acidic conditions. The process is reversible, demonstrating stoichiometric proton–potassium exchange. Taken together, our results map the acid response of K-PHI. They indicate a buffering regime under acidic conditions that is consistent with surface proton adsorption. Further, they identify a protonation and ion exchange threshold at a specific pH (pH 2.0), independent of the ionic strength in solution. Overall, these results provide further insights into the stability window of K-PHI in aqueous dispersion, offering guidance for ion- and pH-sensitive applications such as photocatalysis and beyond.

## Supplementary Material



## Notes

Experimental methods, ICP-AES, EA, TG data, FT-IR and ssNMR spectra, and PXRD patterns (PDF)

## #

Erlangen Center for Interface Research and Catalysis (ECRC), Egerlandstr. 3, 91058 Erlangen, Germany
