# Supplementary material for: From Surface Buffering to Bulk Ion Exchange: Mapping the Acid Response of Potassium Poly(Heptazine Imide) (K-PHI)
Source: Langmuir. 2026 Sep 28;42(40):29595–601. doi: 10.1021/acs.langmuir.6c03166 (PMC13674244; doi:10.1021/acs.langmuir.6c03166)
Supplement: Supplementary file 1 [file la6c03166_si_001.pdf]

SUPPORTING INFORMATION

From Surface Buffering to Bulk Ion Exchange: Mapping the Acid Response of Potassium Poly(heptazine Imide) (K-PHI)

Julia Sinisi,[a,b] Gabriel A. A. Diab,[a,c] Titus Ekabat,[a,d†] Sebastian Bette,[a] Viola Duppel,[a] Ivo F. Teixeira,[c] Stefan Repp,[a] and Bettina V. Lotsch[a,b]***

[a]Nanochemistry Department, Max Planck Institute for Solid State Research, Heisenbergstr. 1, 70569 Stuttgart, Germany.

[b]Department of Chemistry, Ludwig Maximilian University of Munich (LMU), Butenandtstr. 5-13, 81377 Munich, Germany.

[c]Department of Chemistry, Federal University of São Carlos, 13565-905 São Carlos, SP, Brazil.

[d]Department of Physics and Materials Science, Maseno University, Private Bag, 40105 Maseno, Kenya.

*Correspondence: s.repp@fkf.mpg.de

**Correspondence: b.lotsch@fkf.mpg.de

Number of pages: 29

Number of figures: 27

Number of schemes:0

Number of tables: 5

Table of Contents

1. Experimental section 3

Characterization 3

Synthetic procedure 4

K-PHI synthesis 4

K-PHI acid-exposure experimental series 5

K-PHI acidic and alkaline titration 7

K-PHI acidic titration at different K-PHI concentrations with a pH indicator 8

H-PHI reversibility titration 9

2. Supplementary data 11

Acid-exposure series 11

Acid titration curve for different K-PHI concentrations 12

Reversibility titration curve 12

Chemical composition: ICP-AES and EA 13

TGA 15

FT-IR 16

ssNMR 23

PXRD 24

3. References 29

1. Experimental section

Characterization

Inductively coupled plasma atomic emission spectroscopy (ICP-AES) data were obtained from an iCap 6500 instrument (Thermo Fisher Scientific). The sample was digested in HNO_3/HCl using a heating block (Mikroanalytisches Labor Pascher) at 180 °C for 12 h before injection into the plasma.

Elemental analysis (EA) was performed using a Varian Vista-PRO and UNICUBE elemental analyzer system. Samples were digested using a microwave digestion system (SP-D, CEM GmbH) with HNO_3 at 185 °C for 25 minutes, followed by dilution with deionized (DI) water.

Thermogravimetric analysis (TGA) measurements were performed using a STA 449 F3 Jupiter instrument (Netzsch). The samples were analyzed in an Al_2O_3 crucible under an inert atmosphere (Ar flow rate = 70 mL/min), with a heating program ranging from 30 °C to 800 °C at a heating rate of 10 °C/min. Buoyancy correction was applied during the measurements.

Fourier transform infrared spectroscopy (FT-IR) spectra were recorded using an ATR-equipped Spectrum Two spectrometer (PerkinElmer). The data were collected over a range of 4000–400 cm^{-1}, with 25 scans and a spectral resolution of 4 cm^{-1}.

Solid state nuclear magnetic resonance (ssNMR) experiments were performed on a Bruker Neo 600 spectrometer with a magnetic field strength of 14.1 T, corresponding to Larmor frequencies of 600.3 MHz for ^1H and 150.9 MHz for ^{13}C nuclei. All measurements were conducted under Magic Angle Spinning (MAS) conditions to improve spectral resolution, using Bruker BL1.3 and BL3.2 double resonance probes with ZrO_2 rotors of 1.3 mm and 3.2 mm diameter, respectively. For ^1H analysis, direct excitation was employed with 16 scans at MAS frequency of 60 kHz (BL1.3 probe). For ^{13}C, both direct excitation and cross-polarization (CP) techniques were optimized at MAS speed of 20 kHz (BL3.2 probe). Direct excitation was configured for quantitative spectral integration, with a short excitation pulse ($\pi/4$ = 2 μs), a long relaxation delay (60 s), and 1024 scans. CP experiments were conducted with varying contact times (p15 = 1–2 ms) and typically 4000 accumulations. Chemical shifts were referenced externally to tetramethylsilane (TMS, δ = 0.0 ppm) and adamantane as a secondary reference.

Powder X-ray diffraction (PXRD) data were collected using a Stadi P diffractometer (Stoe) in Debye-Scherrer geometry. All samples were packed in 0.5 mm Ø borosilicate glass capillaries (Hilgenberg Glas, 014) and analyzed at room temperature within a 5–50° 2θ range, with a step size of 0.015° and a scan time of 140 s per step. A monochromatic Cu-Kα_1 X-ray source (Ge(111) Johann-type) was employed, and data were detected using a Mythen 1K (DECTRIS) detector.

Transmission electron microscopy (TEM) analysis was carried out using a Philips CM 30 ST microscope operated at 300 kV with a LaB_6 cathode. Bulk samples were finely ground and dispersed onto a holey carbon-coated copper grid. Micrographs were recorded using a TVIPS TemCam-F216 CMOS camera. Fast Fourier Transform (FFT) analyses were performed using the EM-Menu 4.0 Extended software package. Energy-dispersive X-ray spectroscopy (EDX) was conducted in TEM mode with a Noran System Seven (NSS) Si(Li) detector.

Zeta potentials were measured using a Malvern Zetasizer Nano ZS. For sample preparation, 1.5 mg of K-PHI was dispersed in 150 mL of 10.0 mM KCl, resulting in a concentration of 0.01 mg/mL. The background electrolyte without acid addition exhibited a pH of 6.4. Aliquots of 10 mL were transferred into vials, and defined amounts of HCl were added to obtain nominal acid exposures corresponding to pH 1.0, 2.0, 3.0, and 4.0. These values refer to the theoretical pH of the background electrolyte in the absence of K-PHI. Each sample was stirred for 30 min prior to analysis.

Synthetic procedure

K-PHI synthesis

The K-PHI framework was synthesized using the ionothermal method, following the procedure outlined by Schlomberg *et al.*[1] First, melon was prepared through the thermal polycondensation of melamine under an inert argon atmosphere. Specifically, melamine (5.0 g, 39.64 mmol, Roth >99%) was placed in a covered quartz glass boat and heated in a tube furnace from room temperature to 550 °C at a ramp rate of 5 °C/min under a continuous argon flow, with a total heating duration of 12 hours. After cooling the furnace to room temperature, the resulting yellow powder, identified as melon, was collected. The powder was then finely ground using a mortar and pestle and washed four times with ultrapure water (18.2 MΩ·cm, Ultra Pure Systems) *via*

centrifugation at 20000 rpm (35329 RCF) for 20 minutes each. Finally, the material was dried overnight in an oven at 60 ℃.

Next, melon was thermally treated in the presence of KSCN, which supplies potassium ions and promotes structural rearrangement toward the PHI framework. For this, a mixture of the synthesized melon (1.5 g) and of KSCN (3.0 g, 30.08 mmol, Roth >98.5%, used as received), were thoroughly ground in a mortar and transferred to an Al_2O_3 boat covered with a quartz glass lid. The mixture was then heated in a tube furnace under argon flow, with an initial ramp from room temperature to 400 °C at 30 °C min^{-1}, followed by a 1 h hold. The temperature was subsequently increased to 500 °C at the same heating rate and held for 30 min. After cooling to room temperature, the material was washed four times with ultrapure water *via* centrifugation at 20000 rpm (35329 RCF) for 20 minutes. The resulting yellow solid was dried overnight in an oven at 60 ℃.

K-PHI acid-exposure experimental series

First, ultrapure water was purged with Ar for 30 min to remove dissolved CO_2, allowing the pH to stabilize within the neutral range. The pH was then adjusted to approximately pH 6.0, pH 4.0, pH 3.0, pH 2.0 or pH 1.0 using HCl. After acid addition, the total volume amounted to 60 mL. Thereafter, KCl was introduced to obtain final concentrations of 2.0 M, 1.0 M, 0.1 M or a KCl-free solution. Following this, 150.0 mg of K-PHI was added and dispersed. Under continuous stirring, the system was equilibrated for an additional 30 min until the pH reached a stable value. Throughout the entire procedure, the suspension was continuously purged with Ar. The pH was recorded before and after K-PHI dispersion using a pH meter (METTLER TOLEDO, SevenDirect SD20 with an InLab Expert Pro-ISM pH electrode) as summarized in Tab. S1. With this approach, the experimental acid-exposure series differs from the K-PHI titration experiments (see next section), as identical equilibration times and constant total solution volumes were maintained, while only the preset pH was varied.

Table S1. Experimental conditions of HCl and KCl, together with the resulting pH after K-PHI dispersion. Each sample was allowed to stabilize for 30 min after K-PHI dispersion, with each dispersion prepared in a total volume of 60 mL. The data are illustrated in Fig. 2a.

Initial pH at given potassium concentration		Equilibrated pH after K-PHI dispersion
2.0 M KCl	1.1 (±0.002)	1.1 (±0.002)
	1.9 (±0.002)	2.4 (±0.002)
	2.9 (±0.002)	8.8 (±0.002)
	3.9 (±0.002)	9.7 (±0.002)
	6.8 (±0.002)	9.7 (±0.002)
1.0 M KCl	1.1 (±0.002)	1.1 (±0.002)
	2.0 (±0.002)	2.4 (±0.002)
	3.0 (±0.002)	9.1 (±0.002)
	4.0 (±0.002)	10.0 (±0.002)
	6.1 (±0.002)	10.0 (±0.002)
0.1 M KCl	1.2 (±0.002)	1.2 (±0.002)
	2.0 (±0.002)	2.5 (±0.002)
	3.0 (±0.002)	10.0 (±0.002)
	4.0 (±0.002)	10.5 (±0.002)
	6.1 (±0.002)	10.5 (±0.002)
0.0 M KCl	1.2 (±0.002)	1.2 (±0.002)
	2.0 (±0.002)	2.5 (±0.002)
	3.0 (±0.002)	10.8 (±0.002)
	4.0 (±0.002)	11.0 (±0.002)
	6.4 (±0.002)	11.1 (±0.002)

After acid exposure, the PHI particles were separated from the solution and washed with EtOH to remove surface adherent ions: Centrifugation at 20000 rpm (35329 RCF) for 10 minutes was followed by six, four, two or one washing steps for 2.0 M KCl, 1.0 M KCl, 0.1 M KCl, 0.0 M KCl subsets, respectively, to account for increased ion adsorption at higher salt concentrations. The purified product was then dried at 60 °C for 2 h.

K-PHI acidic and alkaline titration

In contrast to the acid exposure experiments described above, a direct acid–base titration of K-PHI was performed. A dispersion of K-PHI (50 mg) in 20 mL of Ar-purged ultrapure water served as the titrand. The dispersion was continuously stirred and maintained under constant Ar purging throughout the experiment. After an initial stabilization period of 30 min, either 1.0 M HCl or 1.0 M KOH was added incrementally to probe both acidic and alkaline directions, with the system allowed to equilibrate for an additional 30 min after each addition. Images of the K-PHI dispersion during titration are shown in Figs. S1 and S2, while the corresponding titration curves are presented in Fig. 1.

The acidic titration curve (Fig. 1c) displays a pronounced buffer region with a half-equivalence point in the alkaline range at pH 10.8, corresponding to an apparent pK_B value of 3.2 according to the Henderson–Hasselbalch equation. However, the applicability of this model to heterogeneous solid–liquid systems is inherently limited, not least because equilibrium establishment cannot be presumed[2, 3] (see discussion in the main text). The equivalence point of the titration curve is calculated to pH 6.6, suggesting completion of the reaction beyond this value. Notably, a distinct color change was observed at the first measurement point beyond the equivalence point (Fig. S1d). At this same point, the experimental titration curve of K-PHI coincides with the theoretical titration line obtained in the absence of K-PHI, indicating that the buffering capacity of K-PHI is fully exhausted and H-PHI has formed (Fig. 1c).

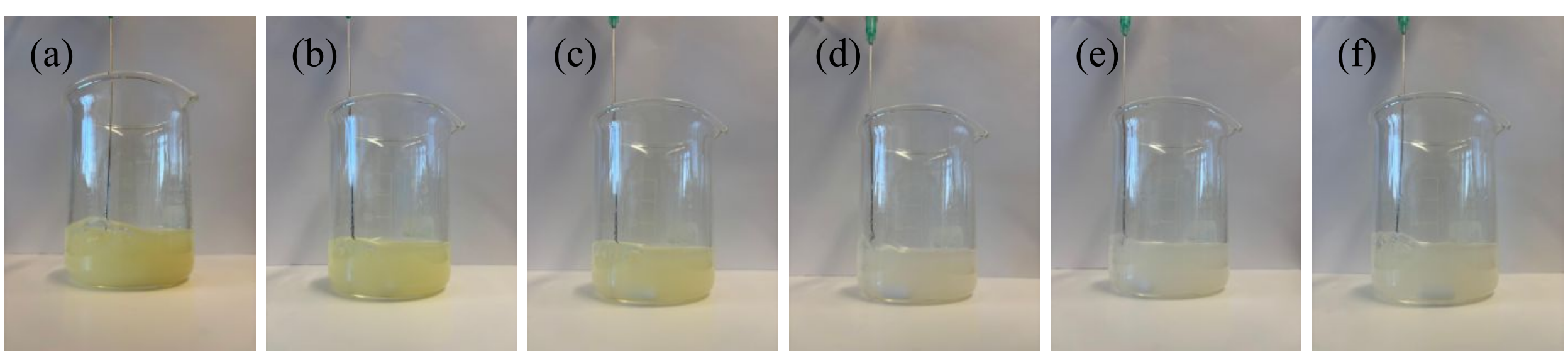

Figure S1. Series of images documenting the titration of K-PHI with 1.0 M HCl: (a) K-PHI dispersed in neutral ultrapure water resulting in pH 10.9, and after incremental acid additons leading to (b) pH 10.8, (c) pH 10.8, (d) pH 3.1, (e) pH 2.5, (f) pH 1.4. Images and pH values were recorded after a stabilization

period of 30 min following each acid addition. For volumes and the corresponding titration curve refer to Fig. 1c. In (d), a color change to white is observed, suggesting the formation of H-PHI.

The alkaline titration of K-PHI with KOH probes the same buffer region, exhibiting a half-equivalence point at pH 10.8 (Fig. 1b), in agreement with the acidic titration. Exhaustion of the buffering capacity under alkaline conditions is indicated by convergence of the experimental titration curve with the theoretical curve obtained in the absence of K-PHI.

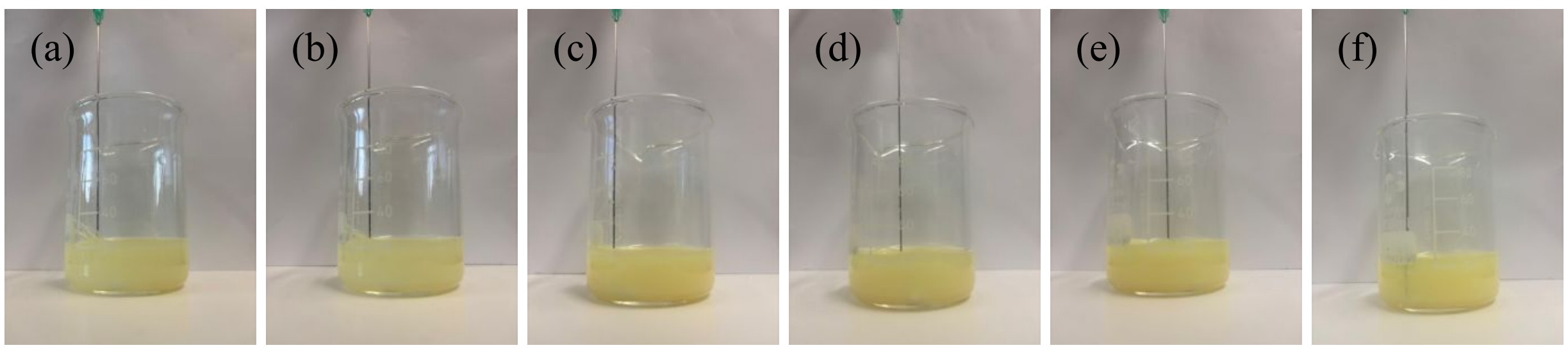

Figure S2. Series of images documenting the titration of K-PHI with 1.0 M KOH: (a) K-PHI dispersed in neutral ultrapure water resulting in pH 10.9, and after incremental base additons leading to (b) pH 10.9, (c) pH 11.0, (d) pH 11.5, (e) pH 11.7, (f) pH 12.7. Images and pH values were recorded after a stabilization period of 30 min following each base addition. For volumes and the corresponding titration curve, including the buffering region, refer to Fig. 1b.

K-PHI acidic titration at different K-PHI concentrations with a pH indicator

Acid titrations were again conducted, but this time at varying K-PHI concentrations and visualized using the pH indicator phenolphthalein. First, 10 mg phenolphthalein were dissolved in 1 mL ethanol. Then, 20 mL Ar-purged ultrapure water were added and the solution was stirred under continuous Ar purging. Different amounts of K-PHI (*i.e.*, 10 mg, 25 mg, 50 mg, 75 mg, 100 mg) were subsequently added, and the suspension was stirred for 30 min to equilibrate before measuring the initial pH. Subsequently, 1 M HCl was added stepwise in µL increments (10 µL, 30 µL, 50 µL, 75 µL, 100 µL, and 200 µL). After each addition, the suspension was equilibrated for 30 min before the pH was measured. Results are illustrated in Fig. S6.

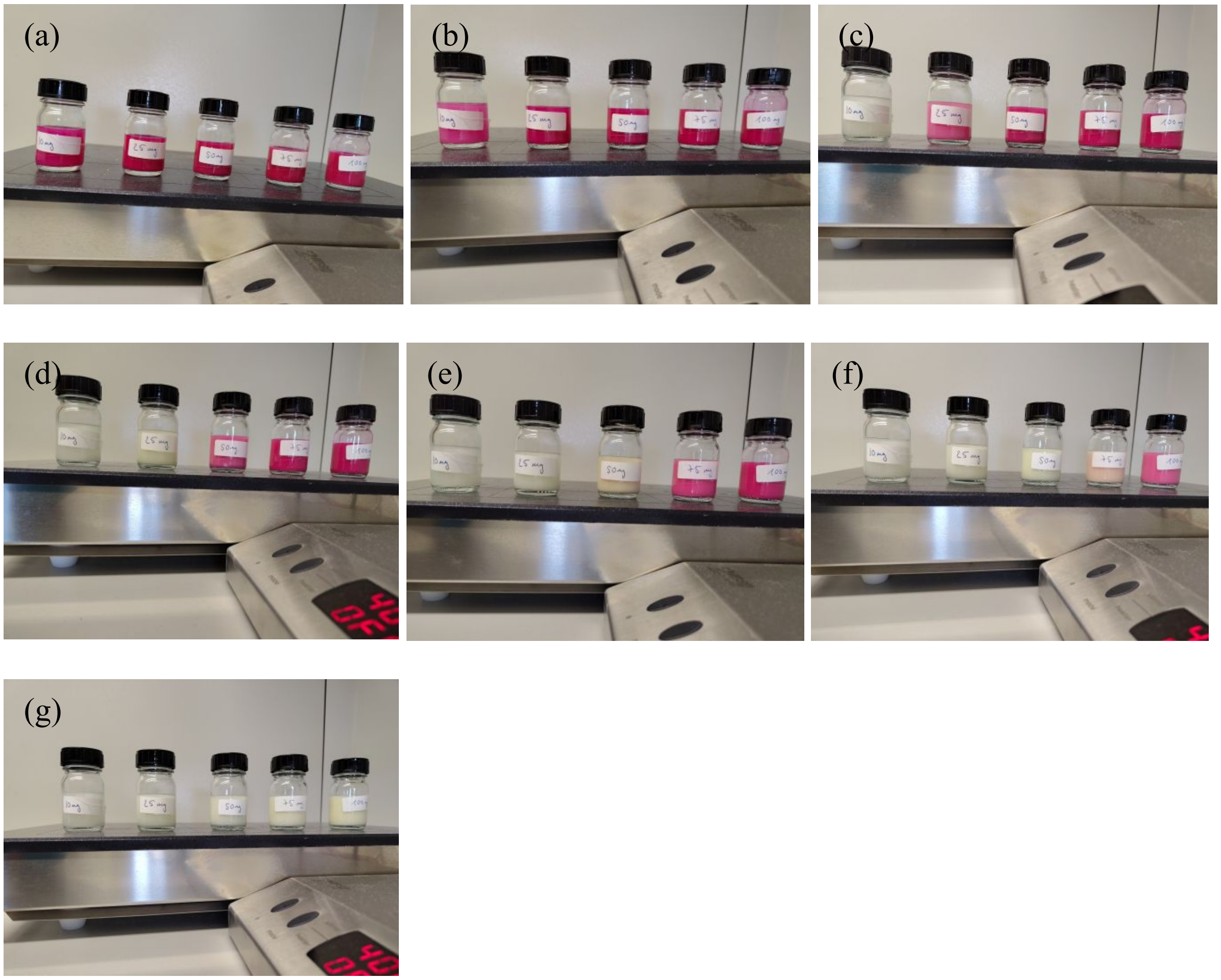

Figure S3. Series of images documenting the titration of K-PHI with 1.0 M HCl and 10 mg phenolphthalein: K-PHI dispersions (a) without acid, and with (b) 10 µL, (c) 30 µL, (d) 50 µL, (e) 75 µL, (f) 100 µL, and (g) 200 µL of HCl added. Images and pH values were recorded after a stabilization period of 30 min following each acid addition. For the corresponding titration curve, refer to Fig. S6.

H-PHI reversibility titration

For the reversibility experiment, H-PHI (50 mg, prepared at pH 2.0 without KCl) was dispersed in 20 mL of Ar-purged ultrapure water. The suspension was continuously stirred and maintained under constant Ar purging throughout the experiment. After an initial stabilization period of 30 min, 1.0 M KOH was added incrementally. Each addition was followed by a 30 min equilibration period, after which the pH was recorded prior to the subsequent addition. Images of

the H-PHI dispersion during titration are shown in Fig. S4, and the corresponding titration curve is presented in Fig. 3a.

The titration curve of H-PHI (Fig. 3a) exhibits a buffer region with a half-equivalence point at pH 4.7, from which an apparent pK_A of 4.7 for H-PHI is derived according to the Henderson–Hasselbalch equation. The lack of complementarity with the pK_B obtained for K-PHI ($pK_A + pK_B \neq 14$) underscores the limited validity of this approach for the present heterogeneous system. The equivalence point calculated from the titration curve is located at pH 8.0 (Fig. 3a). Notably, the onset of the color change from white to pale yellow occurs at pH 11.2 (Fig. S4f), corresponding to the first measurement point beyond the equivalence point and thus consistent with full conversion of the system. The titration was continued until a final pH of 11.6, maximizing the K-PHI yield. Subsequently, the K-PHI particles were separated from solution by centrifugation at 20000 rpm (35329 RCF) for 10 minutes, followed by washing with EtOH at the same speed (20000 rpm, 35329 RCF) for an additional 10 minutes to remove residual ions.

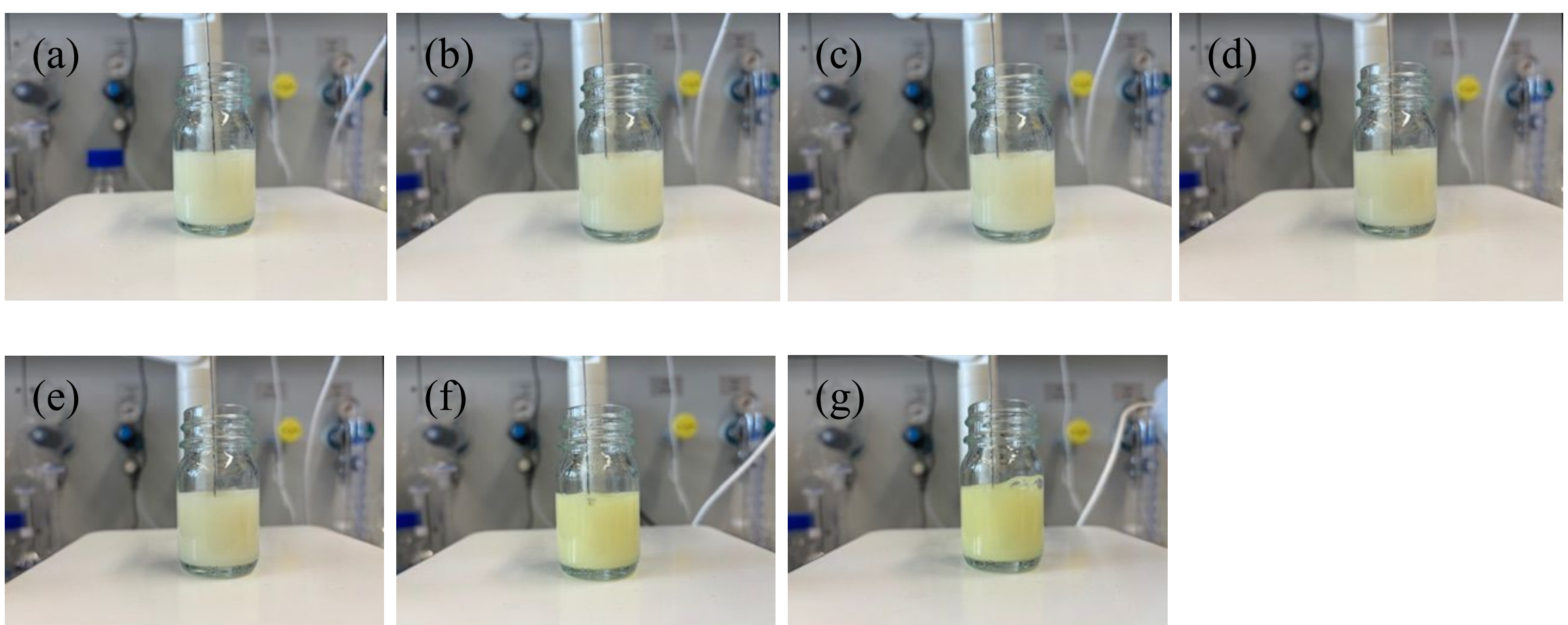

Figure S4. Series of images documenting the titration of H-PHI with 1.0 M KOH: (a) H-PHI dispersed in neutral ultrapure water resulting in pH 4.6, H-PHI dispersions after base additions resulting in (b) pH 4.7, (c) pH 4.9, (d) pH 6.4, (e) pH 8.2, (f) pH 11.2, and (g) pH 11.6. Images and pH values were recorded after a stabilization period of 30 min following each base addition. For volumes and the corresponding titration curve refer to Fig. 3a.

2. Supplementary data

Acid-exposure series

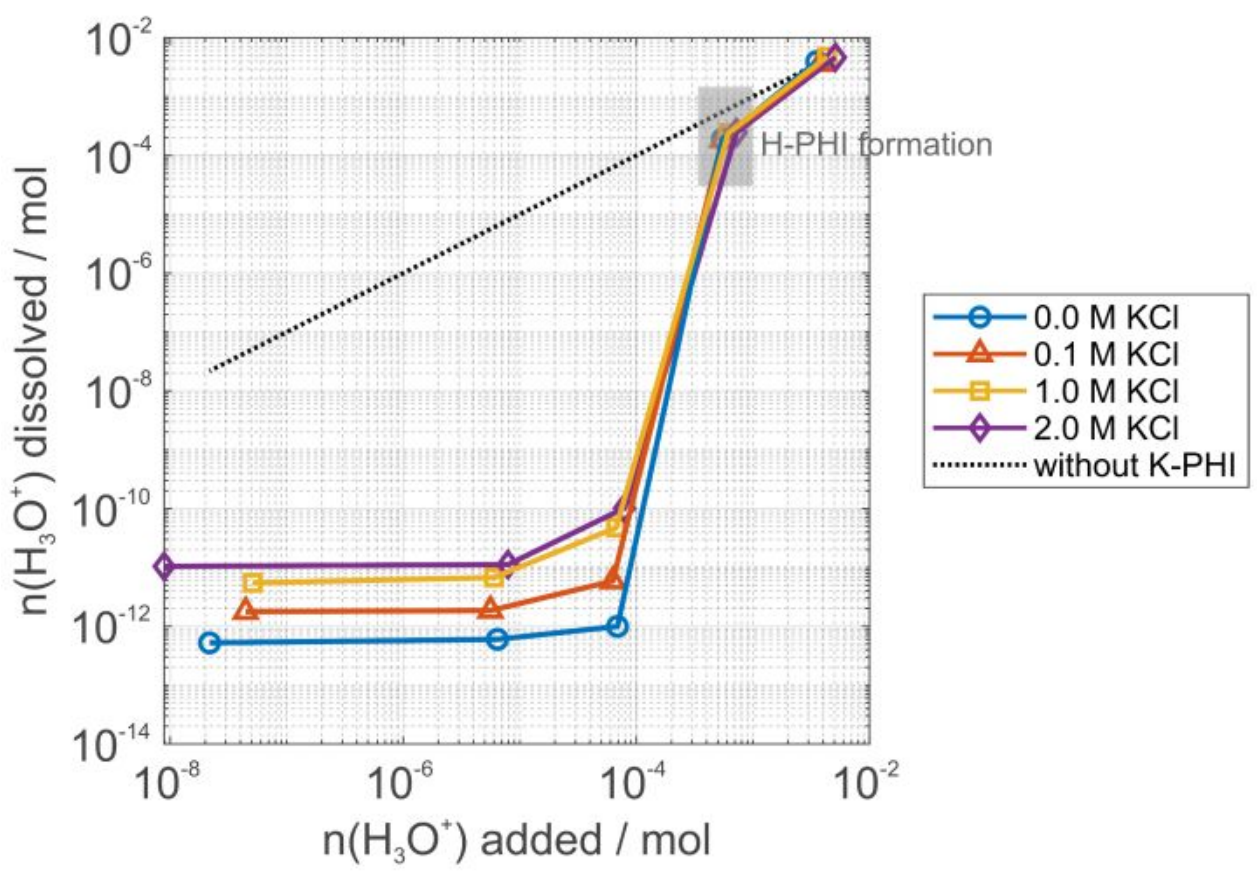

Figure S5. Experimental series on K-PHI dispersions exposed to defined acidic conditions in the absence and presence of varying KCl concentrations. Representation as moles of protons dissolved in solution *vs*. moles of protons added, instead of on a pH scale as in Fig. 2a. Each data point represents a fully characterized sample. Equilibration times and total volumes were identical for all samples to ensure uniform experimental conditions and a constant concentration of solid K-PHI in dispersion, thereby allowing direct comparability. These experiments do not represent titrations; corresponding titration data are shown in Fig. 1. H-PHI formation occurs under sufficiently acidic conditions, *i.e.*, once the alkaline buffering capacity is exceeded (at pH 2.0, see Fig. 2a). The step is characterized by full protonation of the imide bridge (Fig. S11–S15) and the uptake of 0.64 (± 0.02) H^+ per heptazine unit. The same number of protons is released again upon regeneration of K-PHI under alkaline conditions (see Fig. S7).

Acid titration curve for different K-PHI concentrations

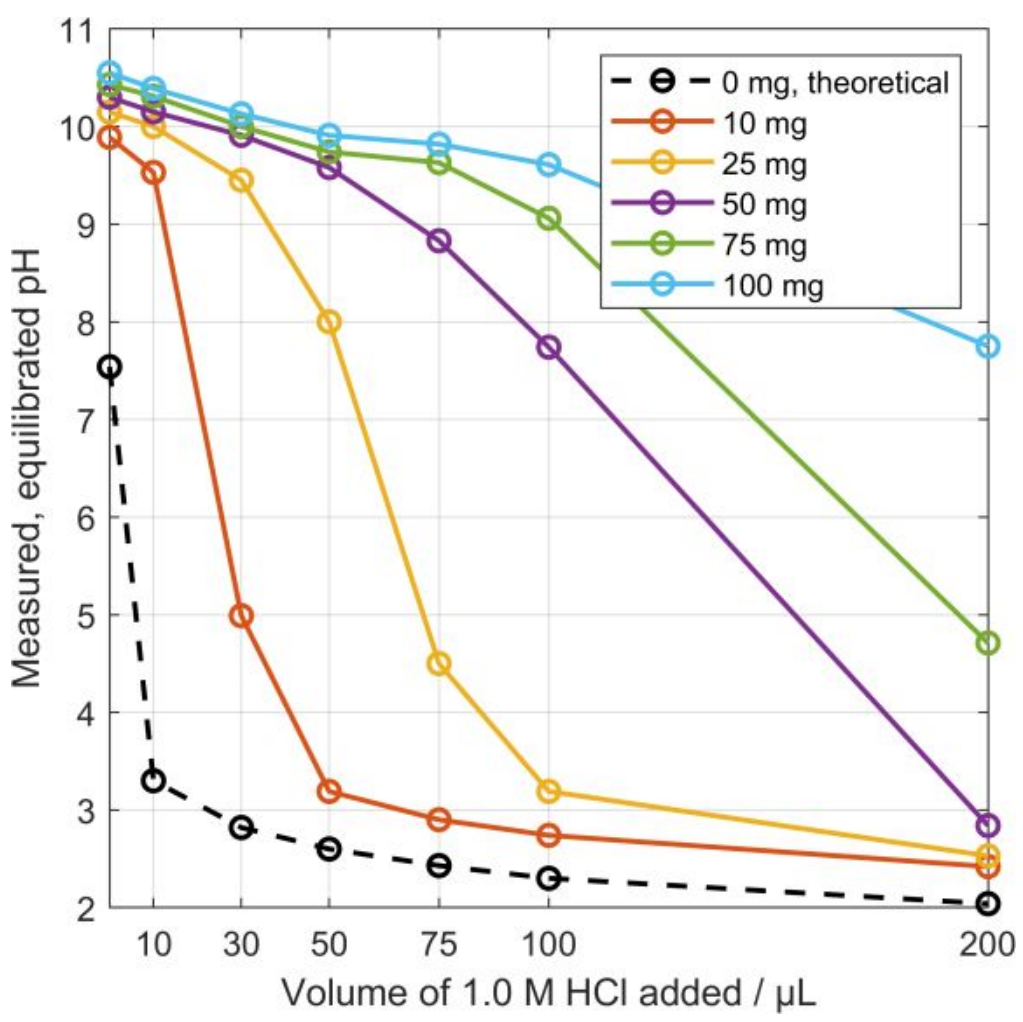

Figure S6. Acidic titration of differently concentrated K-PHI dispersions (10 mg, 25 mg, 50 mg, 75 mg, 100 mg in 20 mL) in the presence of 1 mL phenolphthalein.

Reversibility titration curve

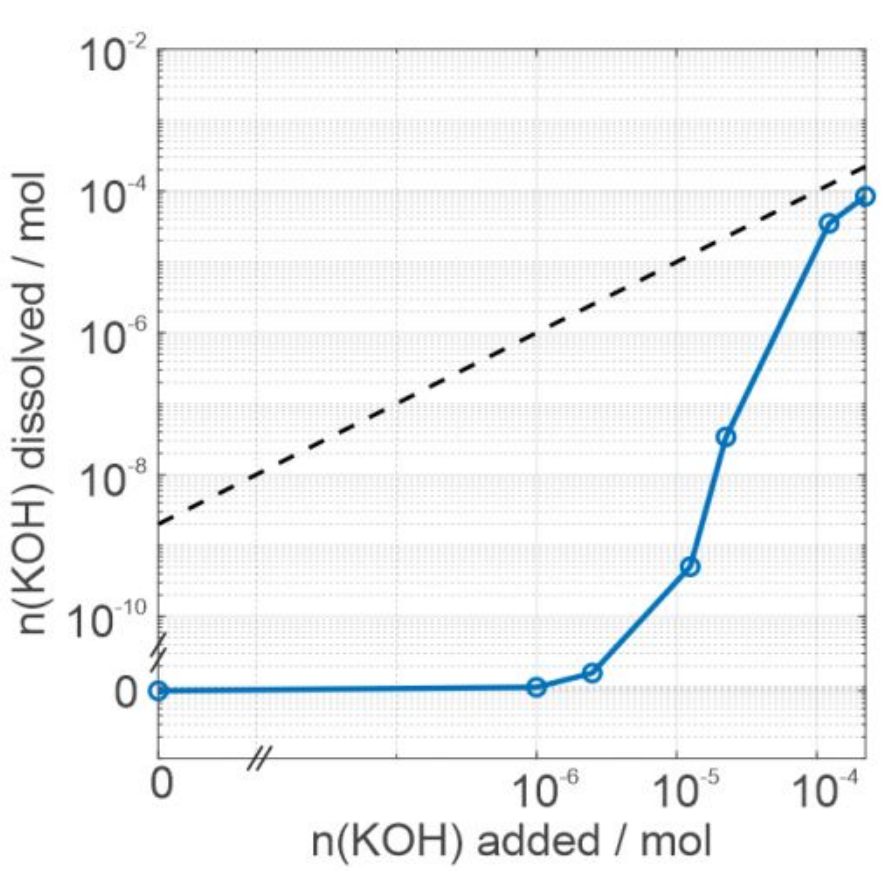

Figure S7. Alkaline titration of H-PHI with 1.0 M KOH. Representation as moles of KOH dissolved in solution *vs*. moles of KOH added, instead of on a pH scale as in Fig. 3a. The final titration step is characterized by complete restoration of potassium interactions at the imide bridges (Fig. S18) and the release of 0.66 (± 0.02) H^+ per heptazine unit, matching the number of protons taken up during protonation (see Fig. S5) and thereby confirming full reversibility of the ion-exchange process. The dashed line represents the theoretical pH in the absence of H-PHI.

Chemical composition: ICP-AES and EA

The values reported in the main text *per heptazine unit* were obtained by dividing the weight percentages by the corresponding molar masses, followed by normalization to nitrogen and conversion to C_7N_{10} units.[1]

Table S2. ICP-AES analysis of potassium in K-PHI exposed to acidic conditions and in K-PHI recovered by H-PHI deprotonation. A graphical summary is provided in Fig. 2b.

**sample condition**	**average K content / wt%**
Pristine K-PHI	12.95 (±0.21)
K-PHI in 2.0 M KCl at pH 1.1	5.07 (±0.03)
K-PHI in 2.0 M KCl at pH 1.9	5.61 (±0.07)
K-PHI in 2.0 M KCl at pH 2.9	11.53 (±0.50)
K-PHI in 2.0 M KCl at pH 3.9	13.60 (±0.00)
K-PHI in 2.0 M KCl at pH 6.8	13.70 (±0.14)
K-PHI in 1.0 M KCl at pH 1.1	4.11 (±0.04)
K-PHI in 1.0 M KCl at pH 2.0	4.71 (±0.02)
K-PHI in 1.0 M KCl at pH 3.0	13.13 (±0.61)
K-PHI in 1.0 M KCl at pH 4.0	13.25 (±0.07)
K-PHI in 1.0 M KCl at pH 6.1	13.35 (±0.07)
K-PHI in 0.1 M KCl at pH 1.2	1.75 (±0.01)
K-PHI in 0.1 M KCl at pH 2.0	2.19 (±0.00)
K-PHI in 0.1 M KCl at pH 3.0	11.00 (±0.14)
K-PHI in 0.1 M KCl at pH 4.0	11.50 (±0.14)
K-PHI in 0.1 M KCl at pH 6.1	12.57 (±0.76)
K-PHI in 0.0 M KCl at pH 1.2	0.52 (±0.04)
K-PHI in 0.0 M KCl at pH 2.0 (H-PHI)	1.36 (±0.00)
K-PHI in 0.0 M KCl at pH 3.0	9.29 (±0.01)
K-PHI in 0.0 M KCl pH 4.0	9.70 (±0.13)
K-PHI in 0.0 M KCl at pH 6.4	9.56 (±0.13)
Regenerated K-PHI	12.30 (±0.14)

Table S3. CHN elemental analysis of K-PHI after KCl-free acid treatment and recovery by alkaline titration. From this, a molar composition of $C_{7.0}N_{10.0}H_{6.6}$ is obtained for regenerated K-PHI.

sample condition	average C content / wt%	average N content / wt%	average H content / wt%	C/N
Pristine K-PHI	27.37 (±0.03)	45.63 (±0.05)	2.09 (±0.01)	0.70
K-PHI in 0.0 M KCl at pH 1.2	29.57 (±0.08)	49.44 (±0.18)	3.13 (±0.05)	0.70
K-PHI in 0.0 M KCl at pH 2.0 (H-PHI)	29.36 (±0.11)	49.10 (±0.09)	3.04 (±0.04)	0.70
K-PHI in 0.0 M KCl at pH 3.0	27.74 (±0.08)	46.26 (±0.25)	2.41 (±0.02)	0.70
K-PHI in 0.0 M KCl at pH 4.0	27.83 (±0.05)	46.43 (±0.01)	2.4 (±0.02)	0.70
K-PHI in 0.0 M KCl at pH 6.4	27.77 (±0.07)	46.23 (±0.02)	2.38 (±0.06)	0.70
Regenerated K-PHI	27.53 (±0.04)	45.65 (±0.09)	2.18 (±0.03)	0.70

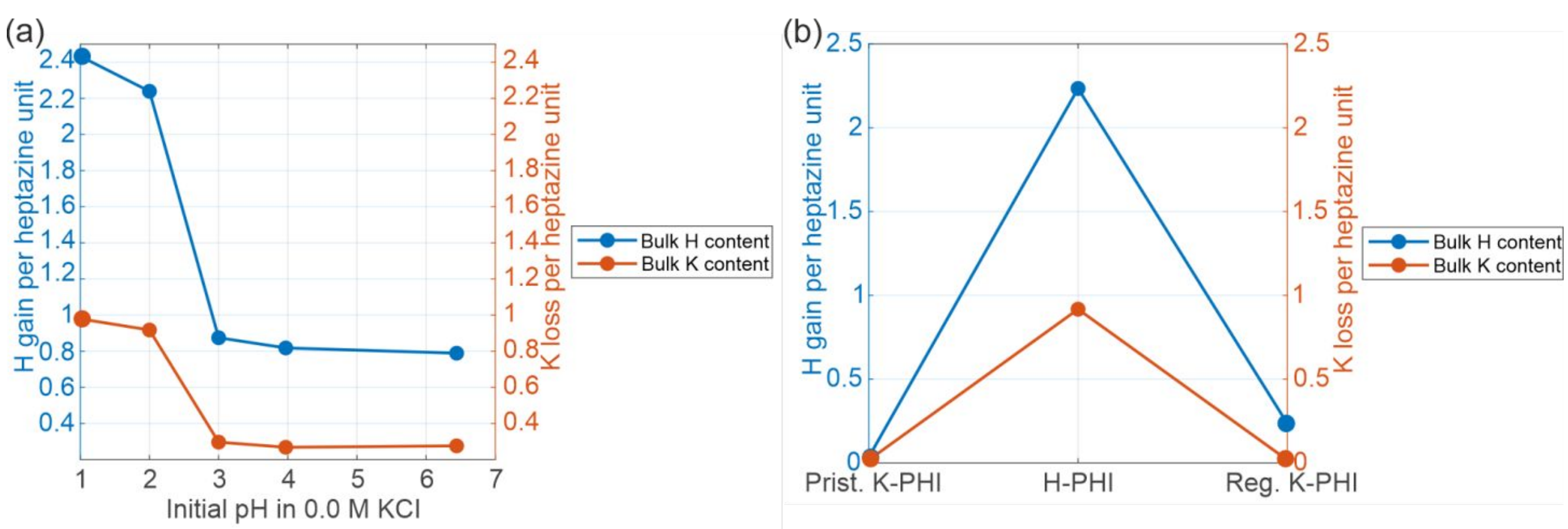

Figure S8. Graphical representation of hydrogen gain, including contributions from H_2O and H_3O^+ species (EA, Tab. S3), and potassium loss (ICP-AES, Tab. S2) for (a) K-PHI under varying acidic conditions and (b) the comparison of pristine K-PHI, H-PHI, and regenerated K-PHI.

Table S4. Potassium ion content according to ICP-AES analysis of K-PHI before and after EtOH washing.

sample condition	average K content / wt%
Pristine K-PHI	11.43 (±0.07)
Pristine K-PHI after EtOH washing	10.99 (±0.10)

TGA

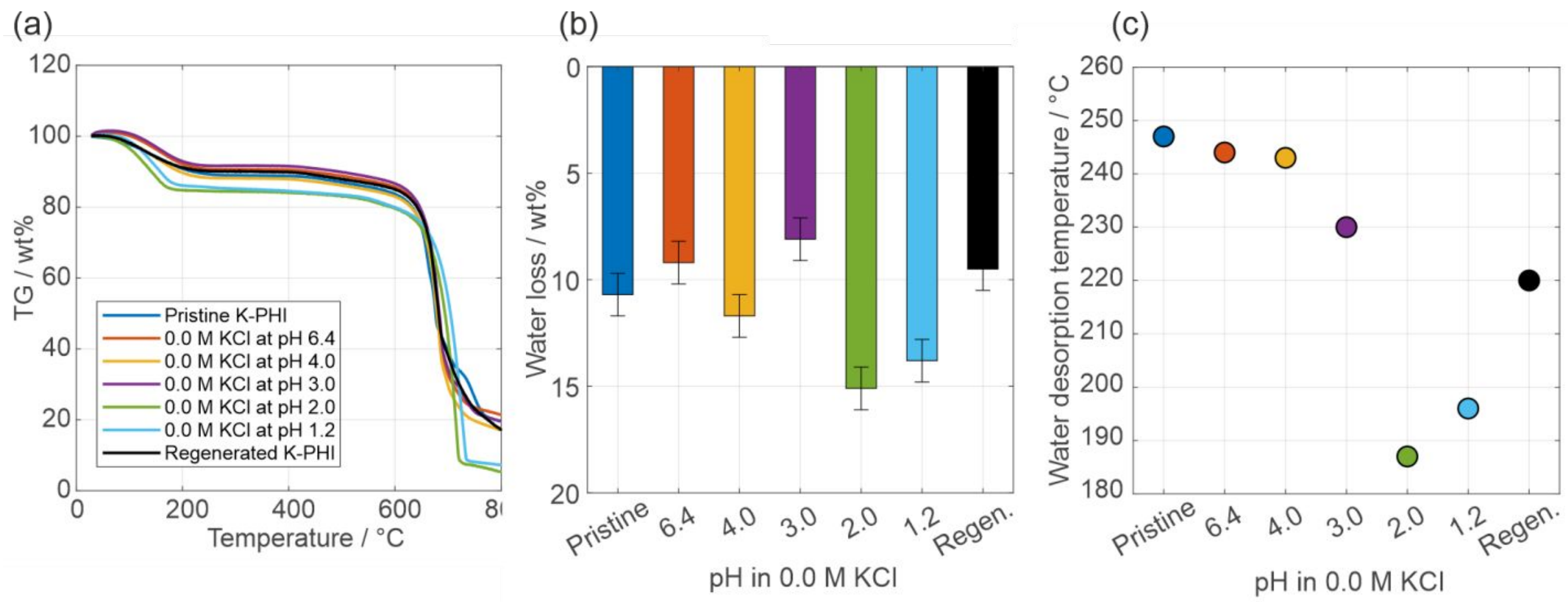

Figure S9. (a) TGA of K-PHI, K-PHI subjected to acidic treatment (KCl-free subset), and K-PHI regenerated from H-PHI. (b) Summary of water losses during the first mass-loss step. Error bars indicate the estimated standard deviation (ESD, 1 wt%). (c) Corresponding desorption temperatures. Samples from pH 2.0 and below are characterized by increased water uptake and reduced desorption temperatures.

FT-IR

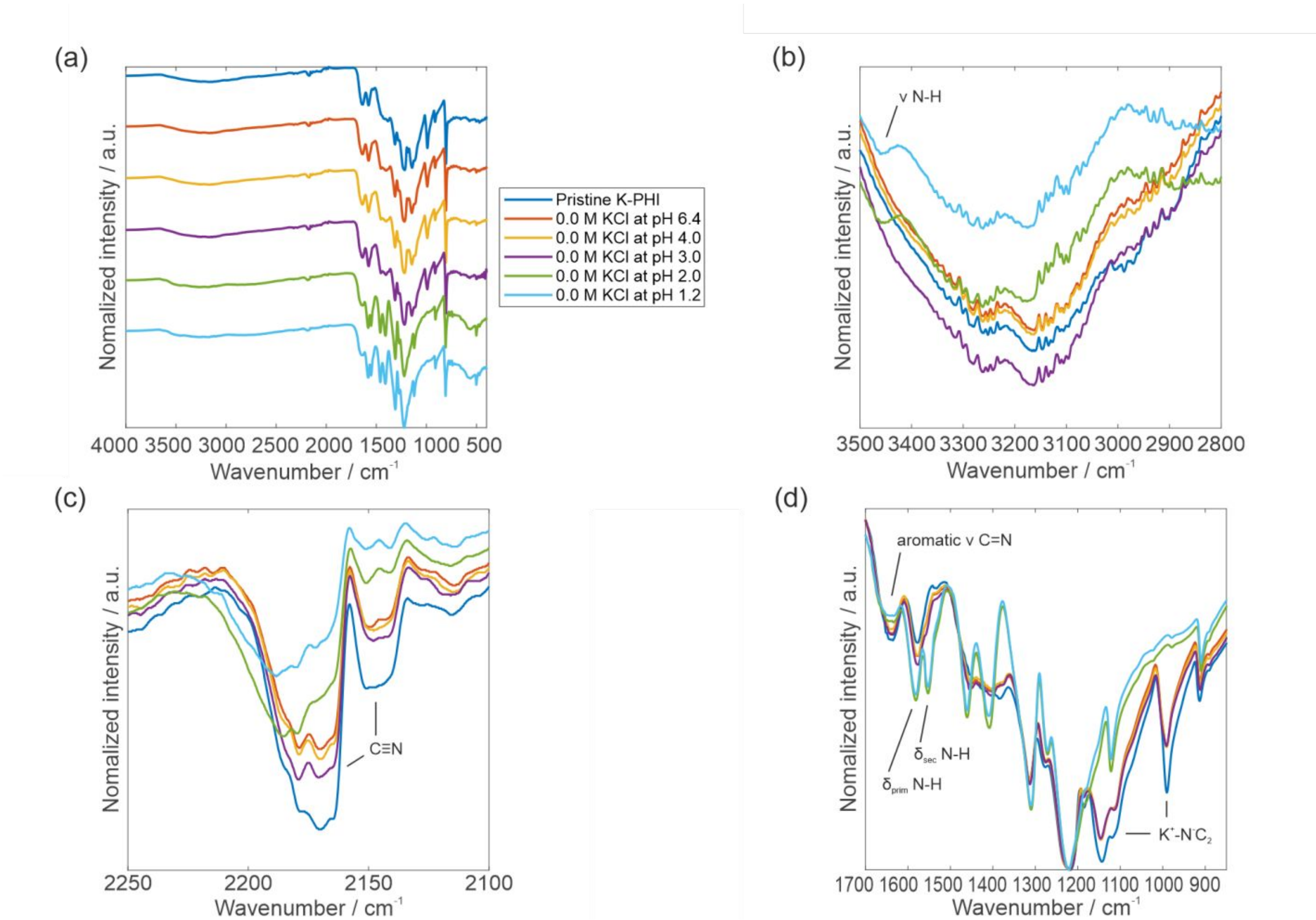

Figure S10. FT-IR spectra of K-PHI under varying pH covering (a) the full range 400–4000 cm⁻¹, with close-ups of (b) 2800–3500 cm⁻¹, (c) the cyanamide region 2100–2250 cm⁻¹, and (d) the fingerprint region 850–1700 cm⁻¹. At pH 2.0, protonation appears as N–H stretching (3450 cm⁻¹) and deformation bands (1580, 1550 cm⁻¹) with concurrent loss of K^+–N^-C_2 interactions (1145, 989 cm⁻¹).[4-6] Protonation of cyanamide groups increases progressively from neutral to acidic conditions, accompanied by a decreasing band intensity at 2170 and 2150 cm⁻¹, underscoring the importance of minimally acidic conditions for H-PHI synthesis. This observation is confirmed by ^{13}C NMR spectra in Fig. S18d.

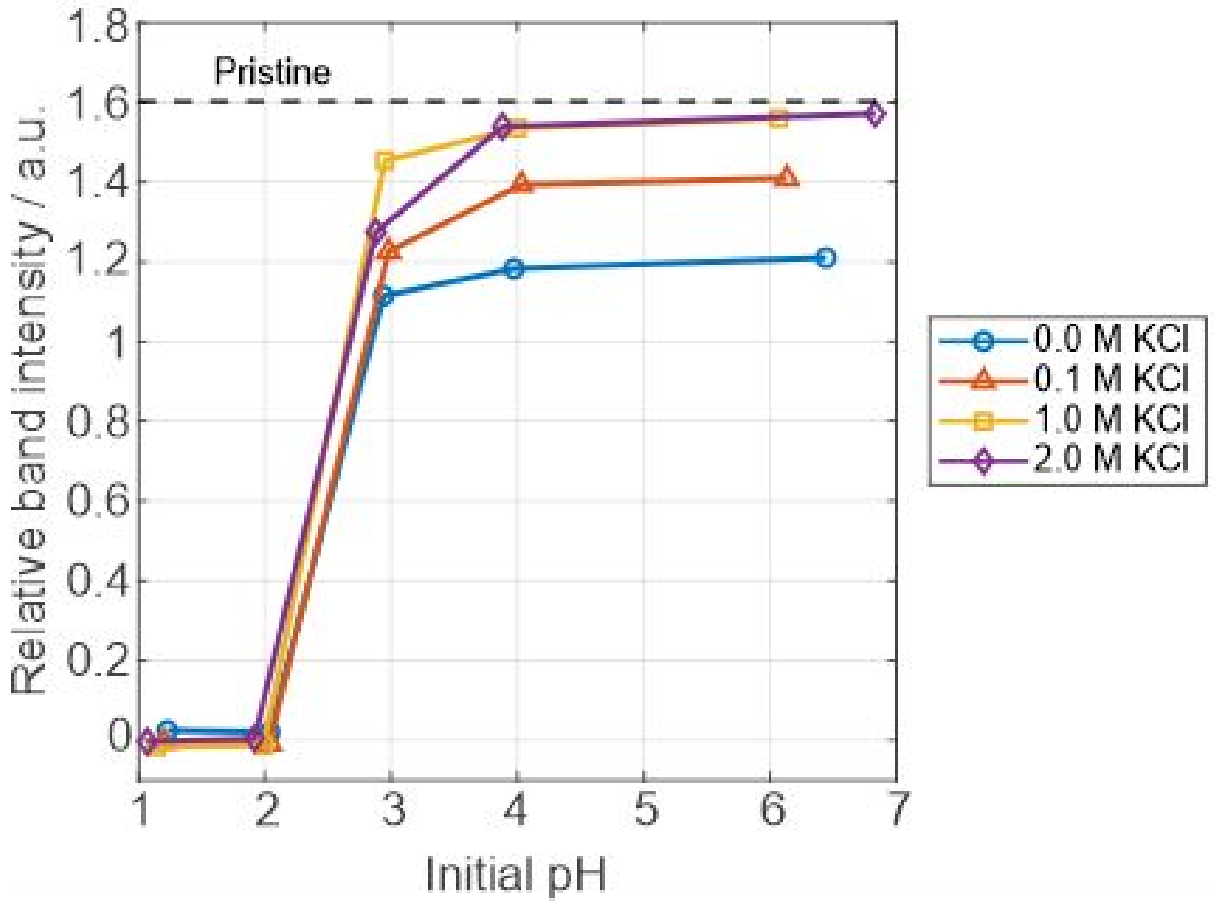

Figure S11. pH-dependent variations in the relative intensity of the 990 cm⁻¹ FT-IR band intensity normalized to 800 cm⁻¹ trace the K^+–N^-C_2 interaction, providing a spectroscopic measure of potassium binding within the carbon nitride backbone.[4-6]

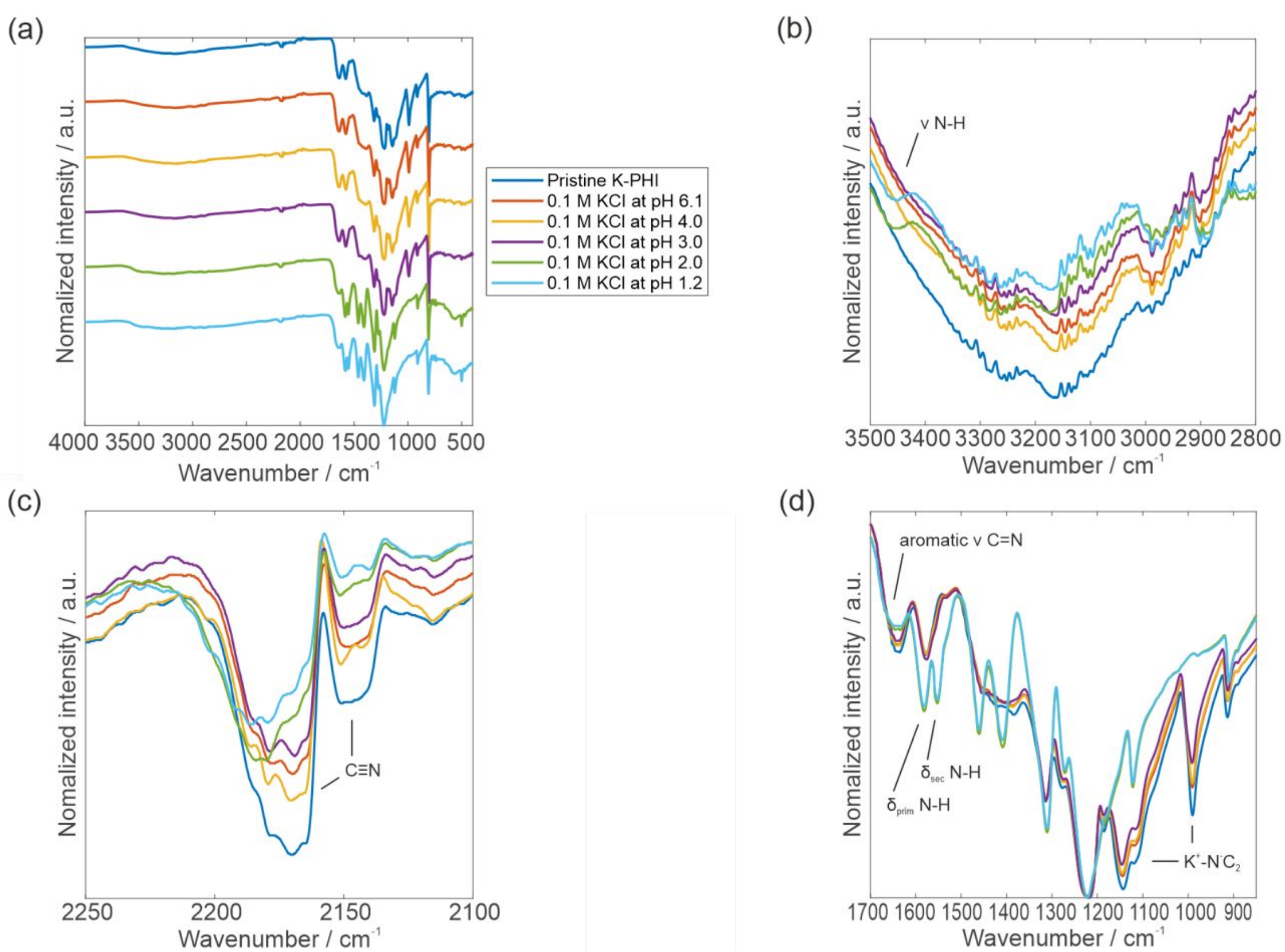

Figure S12. FT-IR spectra of K-PHI in 0.1 M KCl under varying pH covering (a) the full range 400–4000 cm⁻¹, with close-ups of (b) 2800–3500 cm⁻¹, (c) the cyanamide region 2100–2250 cm⁻¹, and (d) the fingerprint region 850–1700 cm⁻¹.

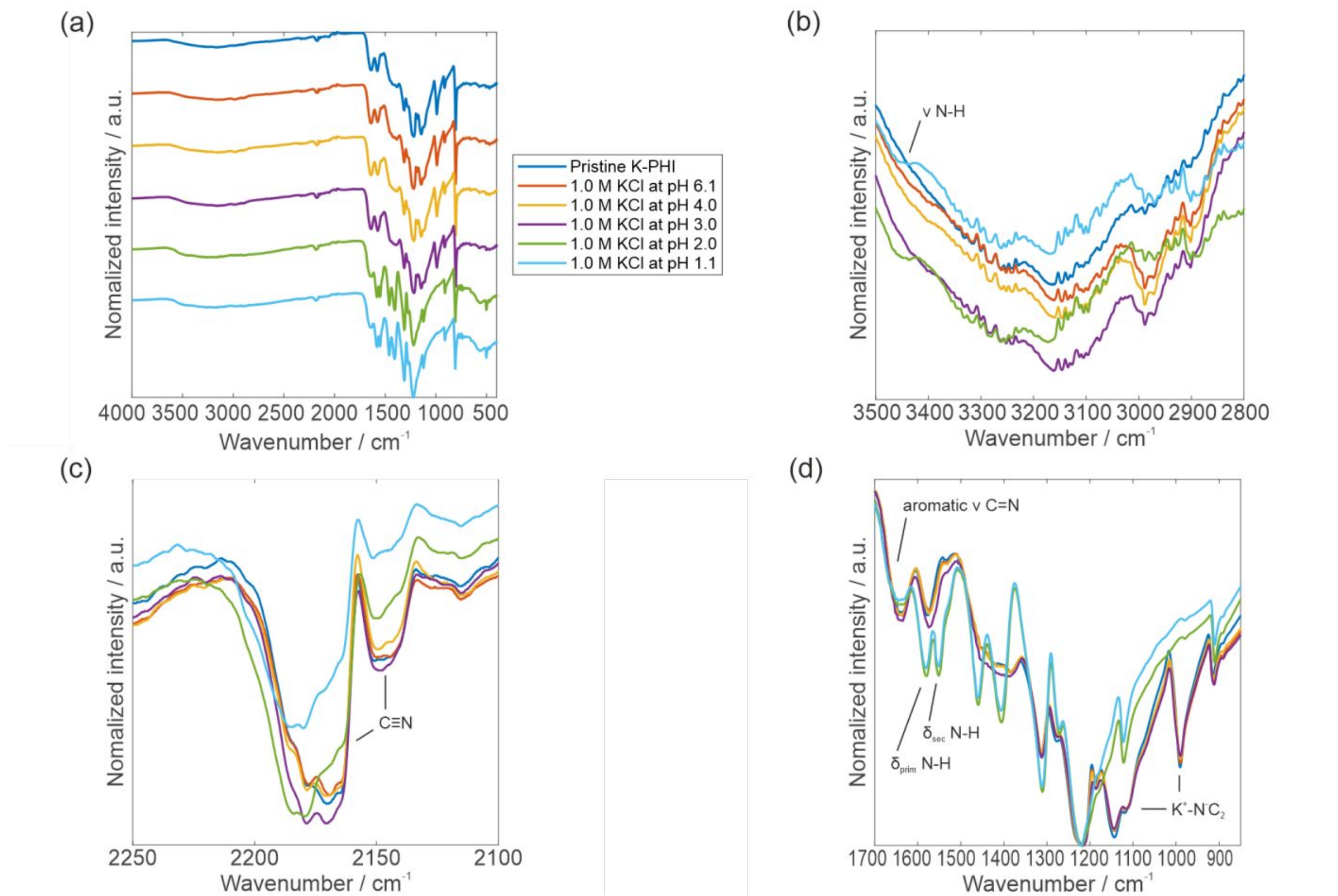

Figure S13. FT-IR spectra of K-PHI in 1.0 M KCl under varying pH covering (a) the full range 400–4000 cm⁻¹, with close-ups of (b) 2800–3500 cm⁻¹, (c) the cyanamide region 2100–2250 cm⁻¹, and (d) the fingerprint region 850–1700 cm⁻¹.

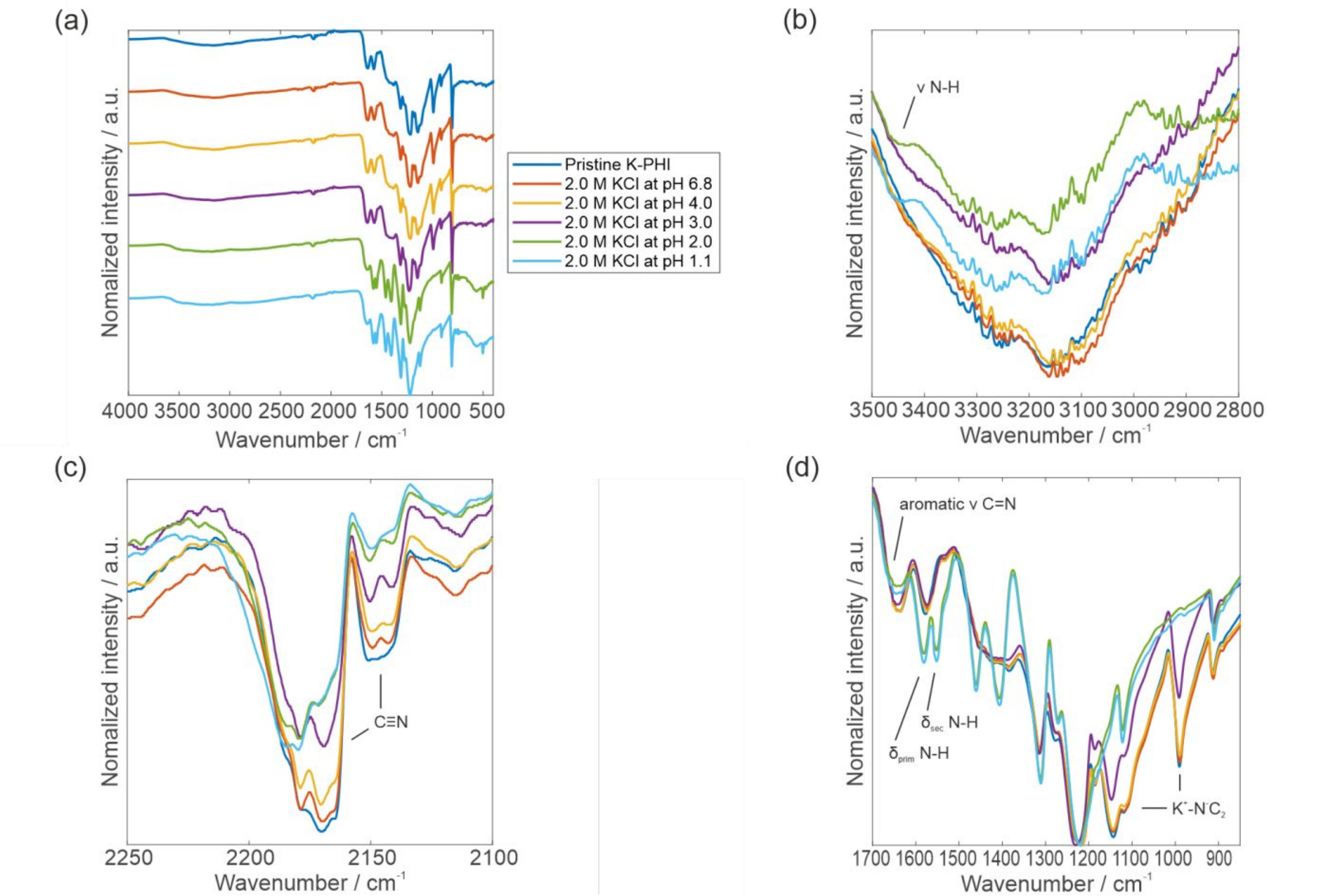

Figure S14. FT-IR spectra of K-PHI in 2.0 M KCl under varying pH covering (a) the full range 400–4000 cm^{-1}, with close-ups of (b) 2800–3500 cm^{-1}, (c) the cyanamide region 2100–2250 cm^{-1}, and (d) the fingerprint region 850–1700 cm^{-1}.

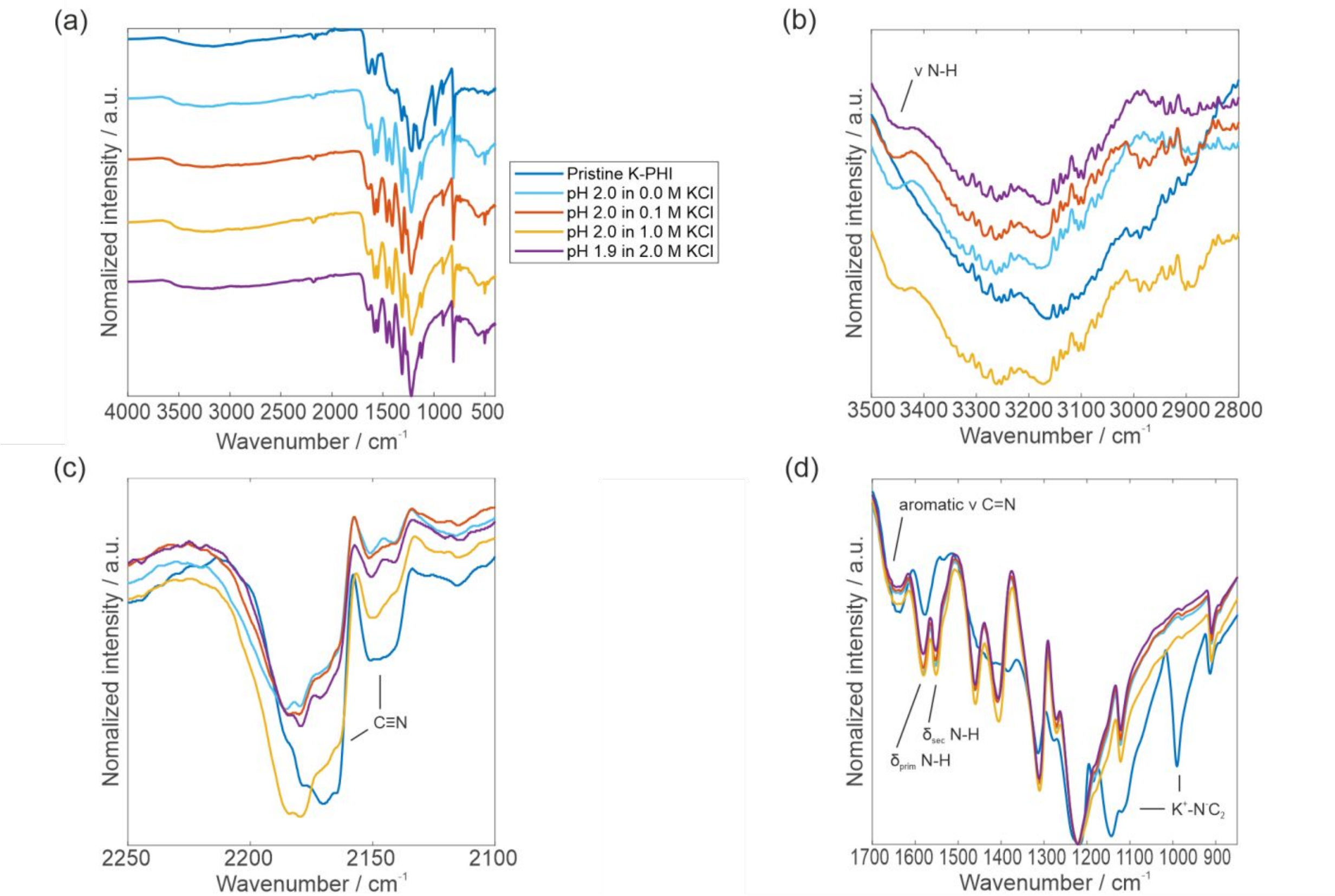

Figure S15. FT-IR spectra of pristine K-PHI and H-PHI obtained from different KCl concentrations, covering (a) 400–4000 cm⁻¹ and close-ups of (b) 2800–3500 cm⁻¹, (c) the cyanamide region 2100–2250 cm⁻¹, and (d) the fingerprint region 850–1700 cm⁻¹. Protonation occurs at pH 2.0 irrespective of the counterion concentration in solution.

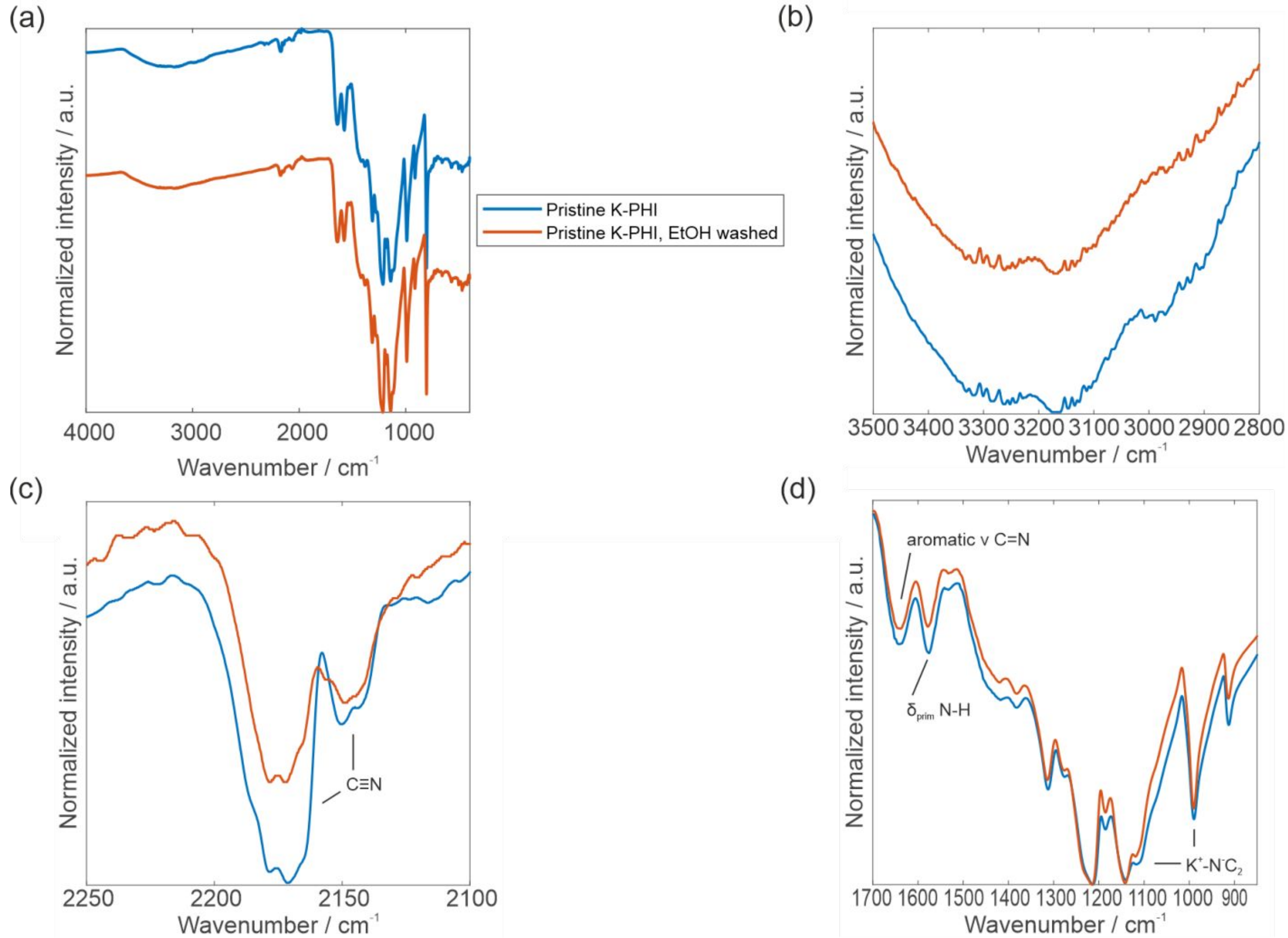

Figure S16. FT-IR spectra of K-PHI before and after EtOH washing in (a) the full range 400–4000 cm^{-1}, with detailed views of (b) 2800–3500 cm^{-1}, (c) 2100–2250 cm^{-1}, and (d) 850–1700 cm^{-1}. The absence of spectral changes confirms that EtOH washing leaves the bonding environment unaffected while enabling surface cleaning.

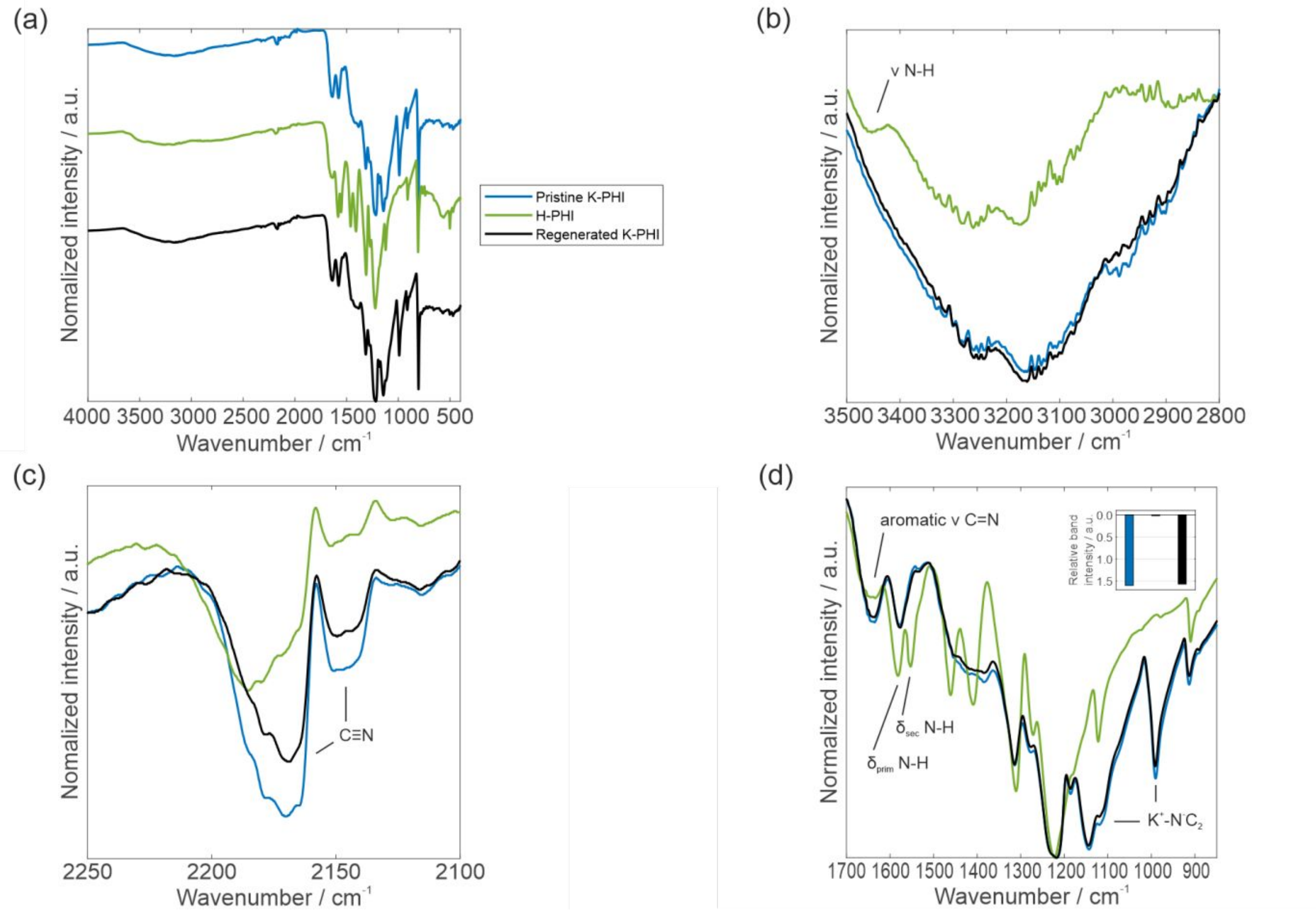

Figure S17. FT-IR spectra of pristine K-PHI, H-PHI, and regenerated K-PHI covering (a) 400–4000 cm⁻¹ with close-ups of (b) 2800–3500 cm⁻¹, (c) 2100–2250 cm⁻¹, and (d) 850–1700 cm⁻¹. The inset in (d) displays the 990 cm⁻¹ band intensity normalized to 800 cm⁻¹, evidencing full reversibility of metal interaction.[4-6]

ssNMR

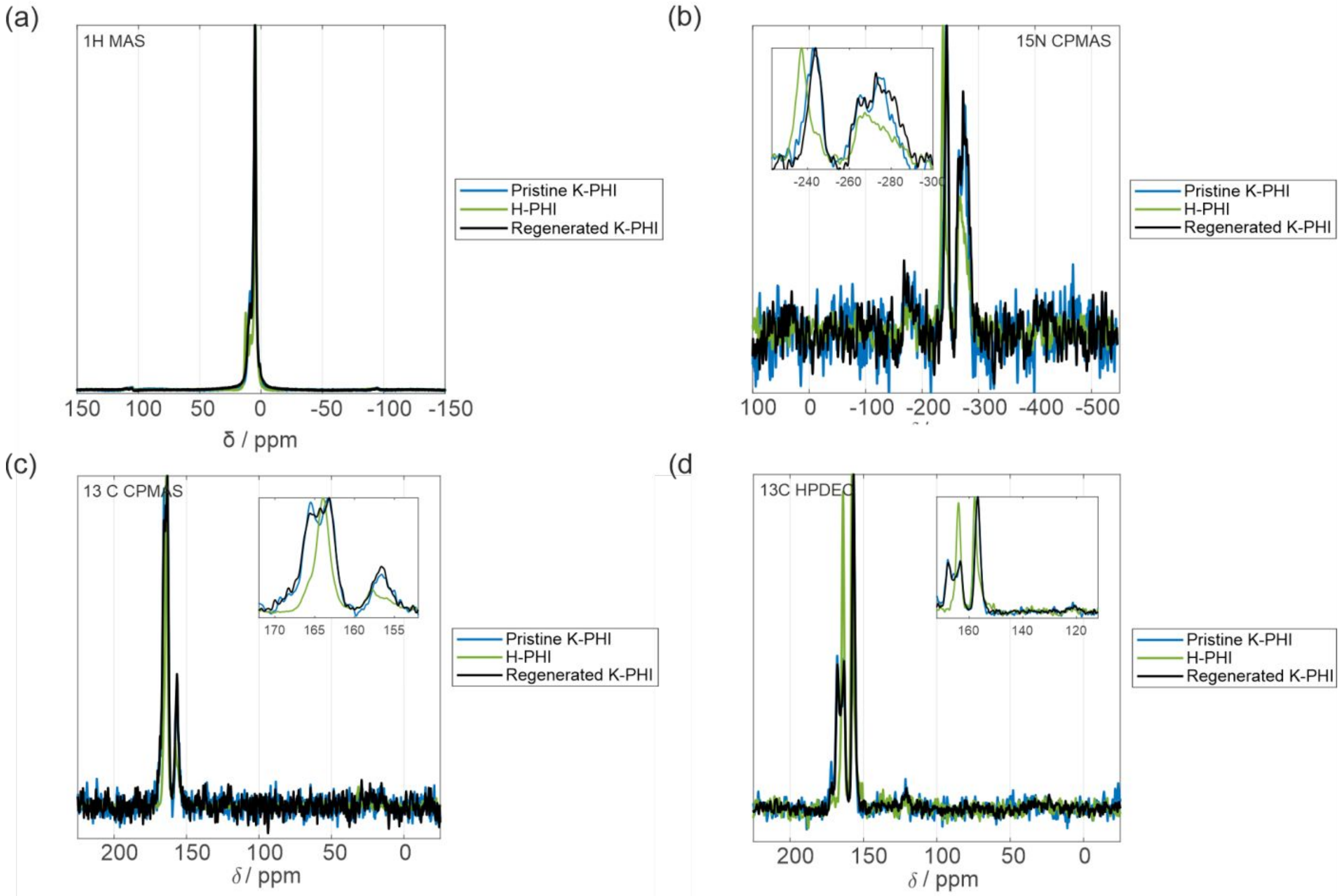

Figure S18. (a) ^{1}H NMR spectra of pristine K-PHI, H-PHI, and regenerated K-PHI. H-PHI shows a downfield-shifted resonance at 12 ppm (compared to 10 ppm in pristine K-PHI; close-up in Fig. 3b), which is assigned to protons in secondary amine groups (imide bridges). This shift consists of deshielding of the NH protons. This shift reflects their increased acidity, as discussed previously by our group.[1] The regenerated K-PHI is characterized by full restoration of the hydrogen atomic organization and bonding environment, as observed in pristine K-PHI. (b) ^{15}N NMR spectra of pristine K-PHI, H-PHI, and regenerated K-PHI. (c) ^{13}C NMR spectra of pristine K-PHI, H-PHI, and regenerated K-PHI. The spectra indicate a more ordered local environment in H PHI compared to K-PHI, and confirms that alkaline titration allows the recovery of a split spectral pattern in regenerated K-PHI consistent with heterogeneous potassium distribution in the layered structure. (d) ^{13}C NMR spectra recorded under high-power proton decoupling (HPDEC) conditions to improve signal resolution. The minor reduction in intensity for H-PHI at 120 ppm aligns with the IR data (Fig. S10, S12–S14), which indicate protonation of K-PHI and cyanamide surface groups under acidic conditions. All assignments follow the literature.[1]

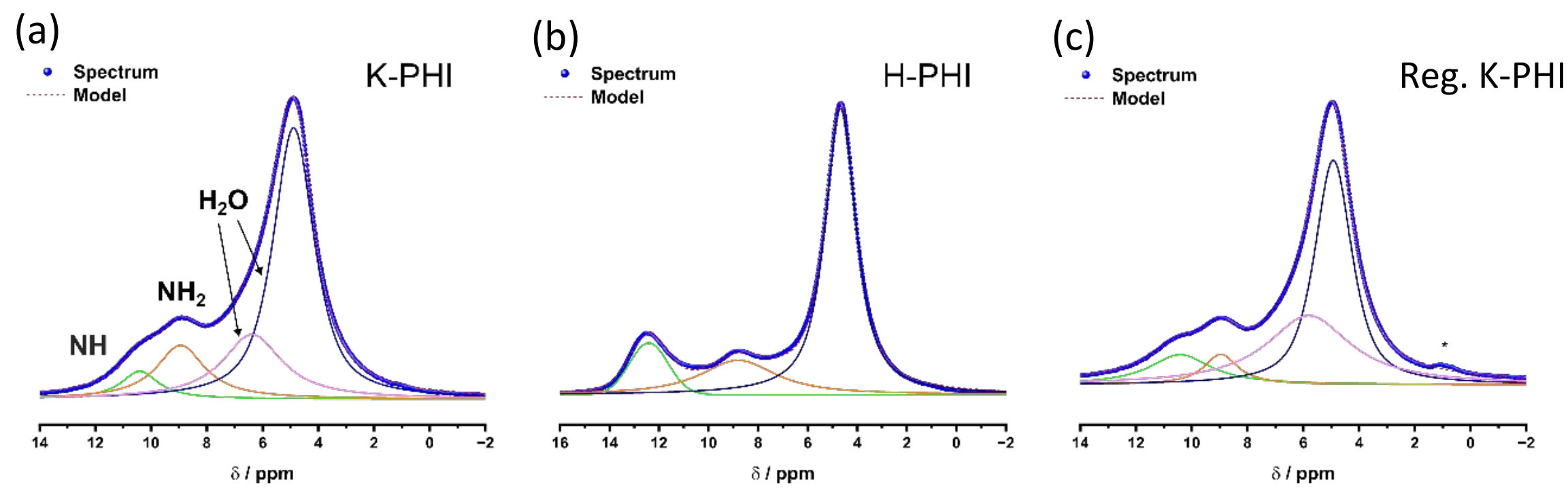

Figure S19. Deconvolution analysis of ^{1}H NMR spectra of (a) pristine K-PHI, (b) H-PHI, (c) regenerated K-PHI.

PXRD

Table S5. Tabulated diffraction angles of the characteristic PXRD peaks of K-PHI after acid exposure and K-PHI recovered from H-PHI by alkaline titration. The FWHM of the 001 reflection, obtained from single line fits, is reported as a measure of coherence along the stacking direction.

sample	2θ of 110 / °	2θ of 010 / °	2θ of 001 / °	FWHM of 001 / °
Pristine K-PHI	7.90	9.92	28.04	0.49
K-PHI in 2.0 M KCl pH 1.1	7.88	10.11	27.93	0.42
K-PHI in 2.0 M KCl pH 1.9	7.88	10.11	27.93	0.42
K-PHI in 2.0 M KCl pH 2.9	7.88	9.90	27.93	0.47
K-PHI in 2.0 M KCl pH 3.9	7.88	9.90	27.93	0.47
K-PHI in 2.0 M KCl pH 6.8	7.88	9.90	27.93	0.48
K-PHI in 1.0 M KCl pH 1.1	7.87	10.11	27.96	0.43
K-PHI in 1.0 M KCl pH 2.0	7.87	10.11	27.96	0.42
K-PHI in 1.0 M KCl pH 3.0	7.91	9.87	27.88	0.44
K-PHI in 1.0 M KCl pH 4.0	7.91	9.87	27.96	0.46

K-PHI in 1.0 M KCl pH 6.1	7.91	9.87	27.96	0.48
K-PHI in 0.1 M KCl pH 1.2	7.86	10.04	28.04	0.43
K-PHI in 0.1 M KCl pH 2.0	7.86	10.04	28.04	0.44
K-PHI in 0.1 M KCl pH 3.0	7.91	9.90	28.03	0.45
K-PHI in 0.1 M KCl pH 4.0	7.90	9.90	28.03	0.47
K-PHI in 0.1 M KCl pH 6.1	7.90	9.90	28.03	0.48
K-PHI in 0.0 M KCl pH 1.2	7.87	10.11	28.03	0.42
K-PHI in 0.0 M KCl pH 2.0 (H-PHI)	7.87	10.11	28.03	0.43
K-PHI in 0.0 M KCl pH 3.0	7.89	9.90	28.03	0.46
K-PHI in 0.0 M KCl pH 4.0	7.89	9.90	28.03	0.46
K-PHI in 0.0 M KCl pH 6.4	7.89	9.90	28.03	0.45
Regenerated K-PHI	7.90	9.92	28.10	0.51

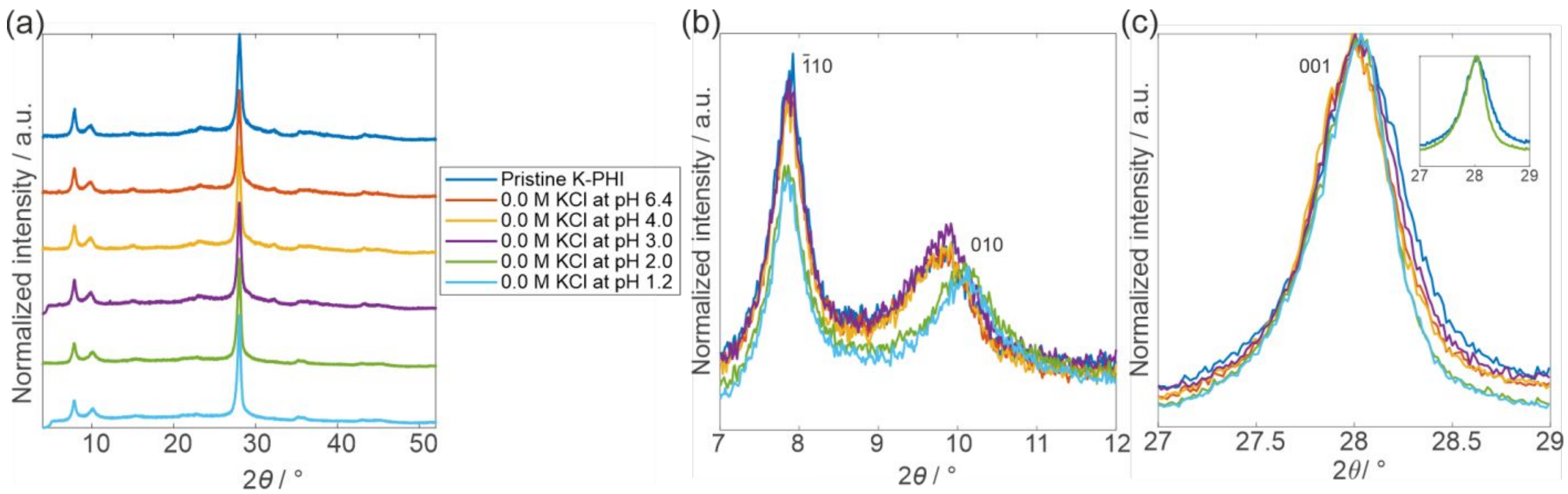

Figure S20. PXRD patterns of K-PHI under varying pH, covering (a) the full 2θ range, with close-ups of (b) the 7–12° 2θ region, and (c) the 27–29° 2θ region. With protonation starting at pH 2.0, the 010 peak at 10° shifts to higher angles, suggesting sheet slipping analogous to cation exchange with smaller metal ions.[7] At the same time, a very slight sharpening of the 001 reflection is observed, indicating a minor increase in coherence in *c*-direction. In PHI materials, both parameters are known to fluctuate depending on the hydration water and potassium.[1] These structural characteristics, in turn, are governed by the preparation

route of H-PHI, particularly the acidic treatment conditions and the nature of the starting material. Accordingly, harsher protonation conditions or the use of more crystalline precursors may result in altered structural responses, as reported in the literature.[1]

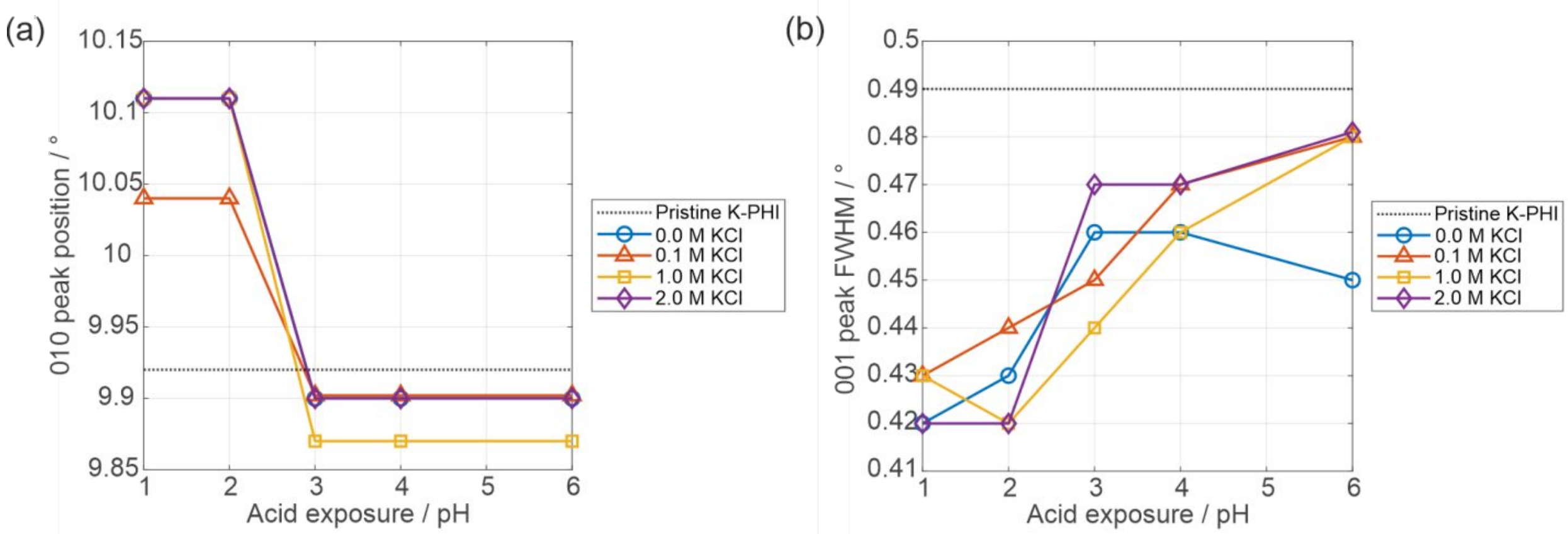

Figure S21. Graphical summary of (a) the 010-peak position (Tab. S5) and (b) the FWHM of the 001 peak (Tab. S5) for K-PHI as a function of acidic pH, showing the trends observed in Fig. S20 to be independent of potassium ion concentration in solution.

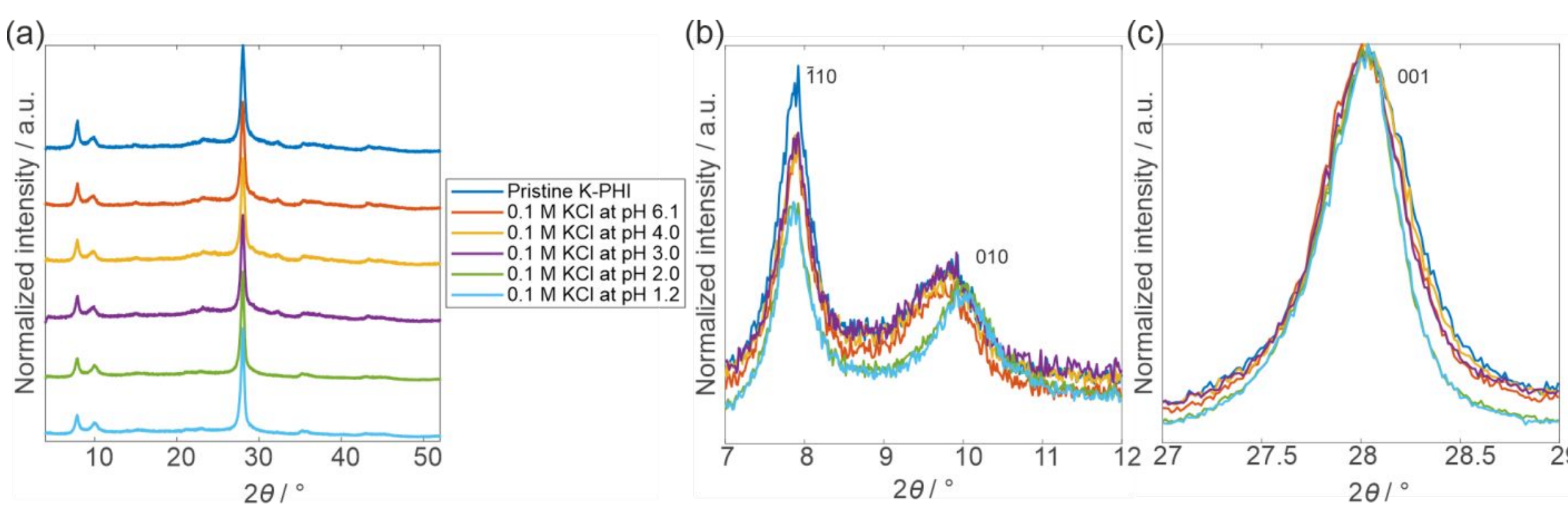

Figure S22. PXRD patterns of K-PHI in 0.1 M KCl under varying pH, covering (a) the full 2θ range, with close-ups of (b) the 7–12° 2θ region, and (c) the 27–29° 2θ region.

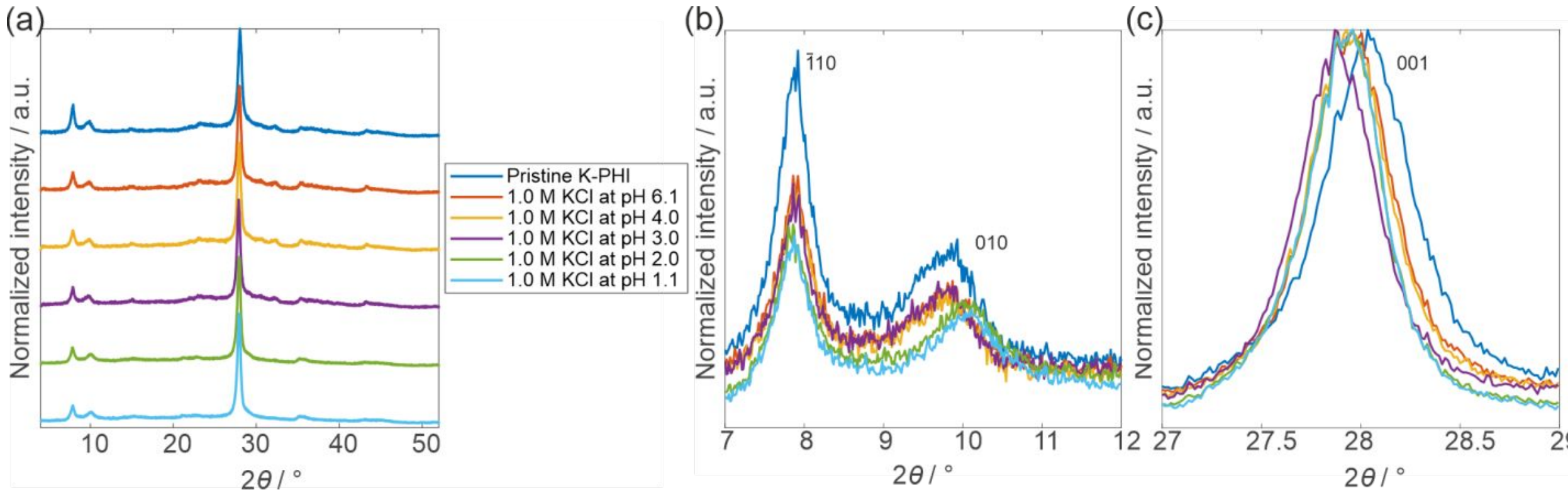

Figure S23. PXRD patterns of K-PHI in 1.0 M KCl under varying pH, covering (a) the full 2θ range, with close-ups of (b) the 7–12° 2θ region, and (c) the 27–29° 2θ region.

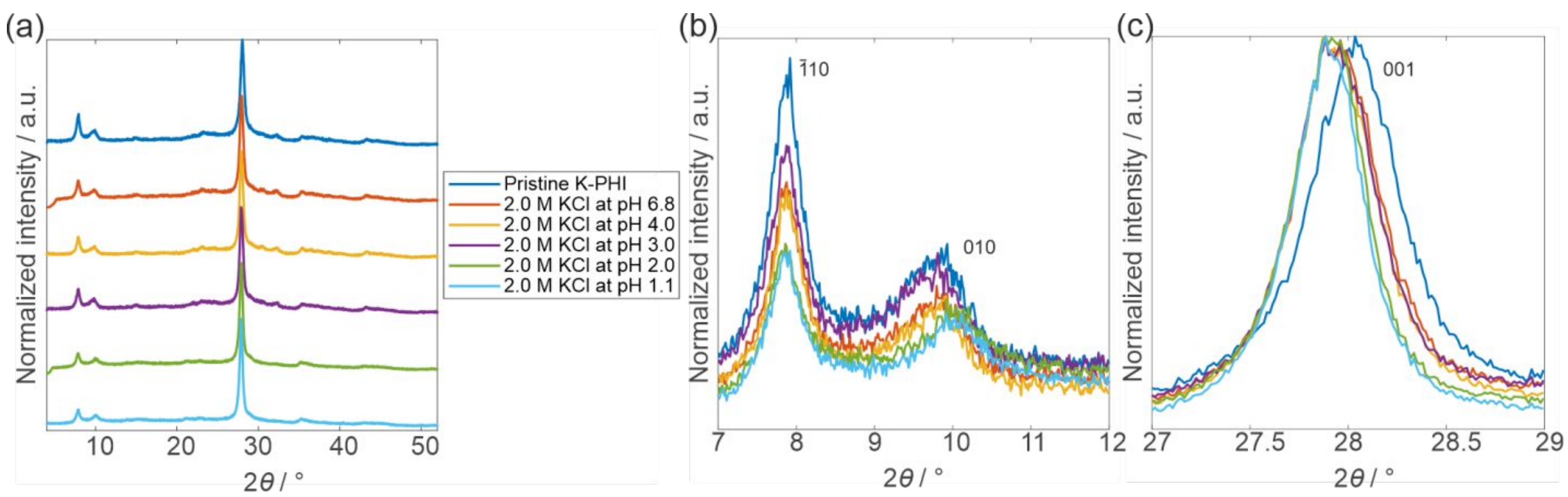

Figure S24. PXRD patterns of K-PHI in 2.0 M KCl under varying pH, covering (a) the full 2θ range, with close-ups of (b) the 7–12° 2θ region, and (c) the 27–29° 2θ region.

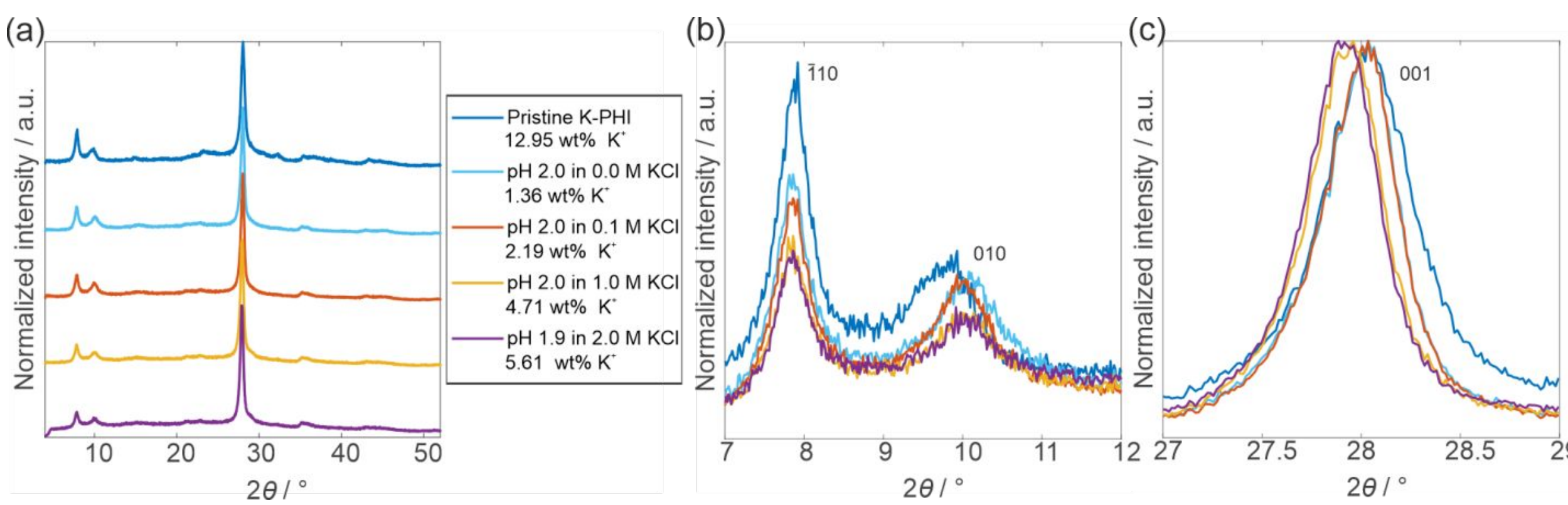

Figure S25. PXRD patterns of pristine K-PHI and H-PHI obtained from different KCl concentrations, covering (a) the full 2θ range, and close-ups of (b) 7–12°, and (c) 27–29°. A shift of the 001 stacking reflection to lower angles is observed, correlating with increasing residual potassium in the material. This

observation is consistent with literature reports describing larger interlayer repeat distances in the presence of potassium and its associated hydration sphere.[1, 7]

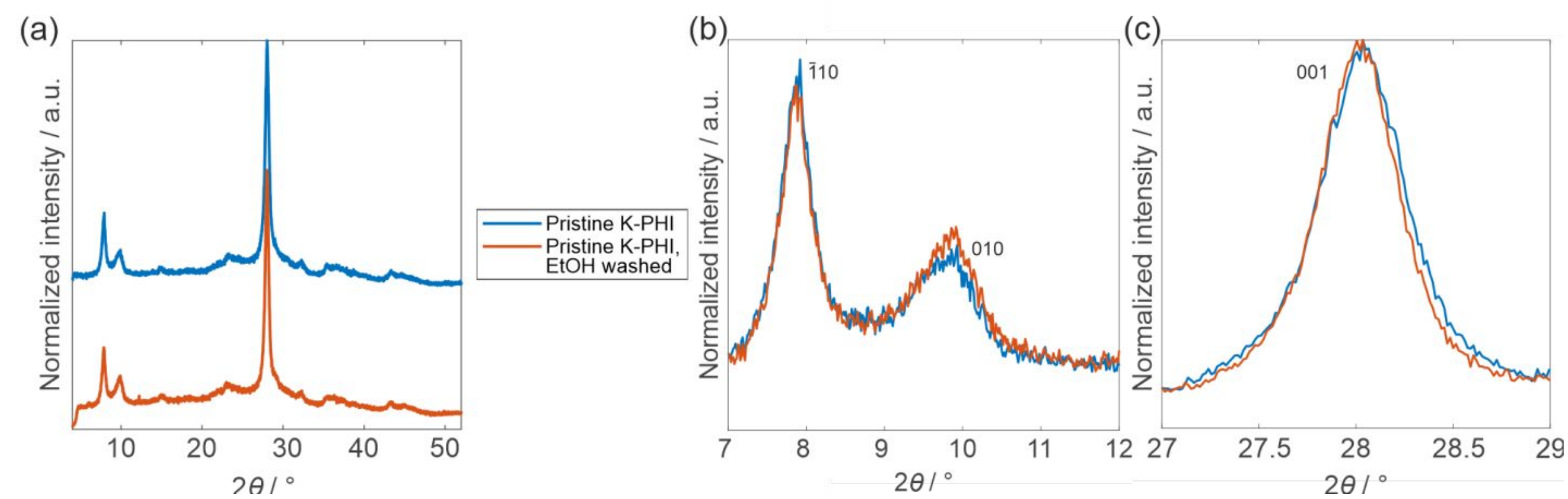

Figure S26. PXRD patterns of K-PHI before and after EtOH washing in (a) the full 2θ range, with close-ups of (b) 7–12° and (c) 27–29°. The absence of peak shifts or broadening confirms that EtOH washing does not alter the crystalline framework while effectively cleaning the particle surface.

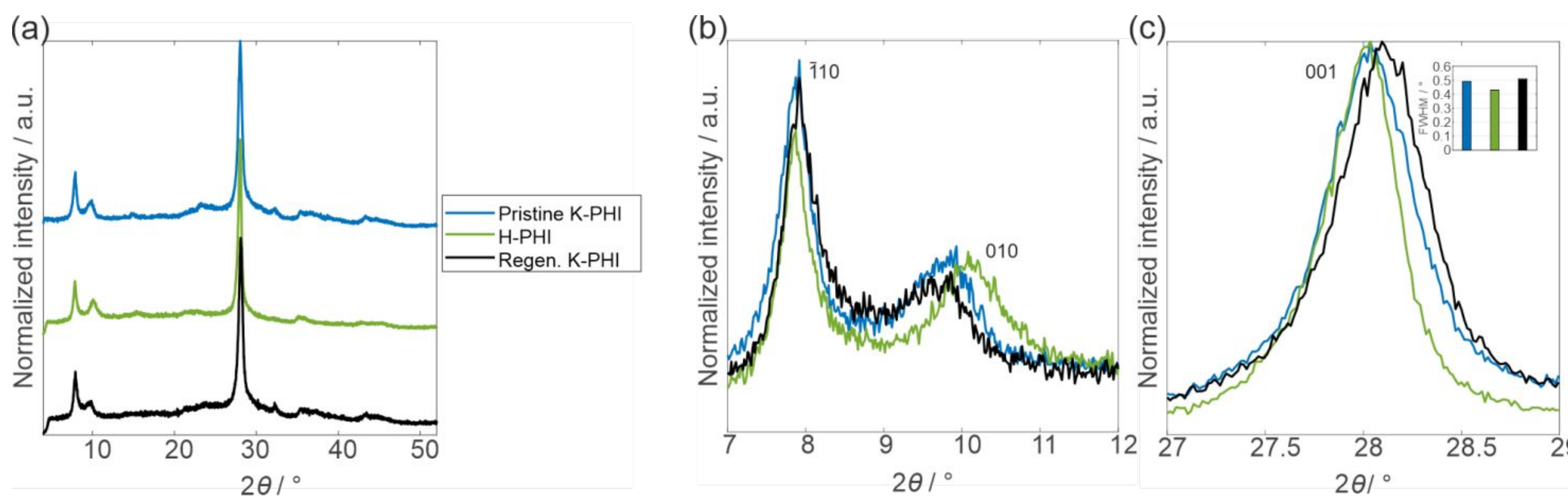

Figure S27. PXRD patterns of pristine K-PHI, H-PHI, and regenerated K-PHI: (a) full 2θ range, (b) 7–12° evidencing reversible sheet slipping, and (c) 27–29° demonstrating batch-to-batch reproducible slightly improved stacking regularity in regenerated K-PHI compared to pristine K-PHI. Thus, the structural changes observed here are reversible and reflect the previously outlined dependence on protonation conditions and starting material characteristics (Fig. S20).

3. References

(1) Schlomberg, H.; Kröger, J.; Savasci, G.; Terban, M. W.; Bette, S.; Moudrakovski, I.; Duppel, V.; Podjaski, F.; Siegel, R.; Senker, J.; Dinnebier, R. E.; Ochsenfeld, C.; Lotsch, B. V. Structural insights into poly (heptazine imides): a light-storing carbon nitride material for dark photocatalysis. *Chem. Mater.* **2019**, *31* (18), 7478–7486.

(2) Corma, A. Inorganic Solid Acids and Their Use in Acid-Catalyzed Hydrocarbon Reactions. *Chem. Rev.* **1995**, *95* (3), 559–614.

(3) Radić, N.; Prkić, A. Historical remarks on the Henderson-Hasselbalch equation: its advantages and limitations and a novel approach for exact pH calculation in buffer region. *Rev. Anal. Chem.* **2012**, *31* (2), 93–98.

(4) Antonietti, M.; Savateev, O.; Lotsch, B. V. In *Carbon Nitrides: Structure, Properties and Applications in Science and Technology*, Savateev, O., Antonietti, M., Wang, X. Eds.; De Gruyter: Berlin, 2023; pp 1–36.

(5) Bradley, D. C.; Gitlitz, M. H. Metallo-organic compounds containing metal–nitrogen bonds. Part VI. Infrared and nuclear magnetic resonance of dialkylamidoderivatives of titanium, vanadium, zirconium, niobium, hafnium, tantalum, and thorium. *J. Chem. Soc. A* **1969**, 980–984.

(6) Savateev, A.; Pronkin, S.; Willinger, M. G.; Antonietti, M.; Dontsova, D. Towards Organic Zeolites and Inclusion Catalysts: Heptazine Imide Salts Can Exchange Metal Cations in the Solid State. *Chem. Asian J.* **2017**, *12* (13), 1517–1522.

(7) Kröger, J.; Podjaski, F.; Savasci, G.; Moudrakovski, I.; Jiménez-Solano, A.; Terban, M. W.; Bette, S.; Duppel, V.; Joos, M.; Senocrate, A.; Dinnebier, R.; Ochsenfeld, C.; Lotsch, B. V. Conductivity mechanism in ionic 2D carbon nitrides: from hydrated ion motion to enhanced photocatalysis. *Adv. Mater.* **2022**, *34* (7), 2107061.
